# The Msh5 complex shows homeostatic localization in response to DNA double-strand breaks in yeast meiosis

**DOI:** 10.3389/fcell.2023.1170689

**Published:** 2023-05-18

**Authors:** Miki Shinohara, Akira Shinohara

**Affiliations:** ^1^ Department of Advanced Bioscience, Graduate School of Agriculture, Kindai University, Nara, Japan; ^2^ Agricultural Technology and Innovation Research Institute, Kindai University, Nara, Japan; ^3^ Institute for Protein Research, Osaka University, Osaka, Japan

**Keywords:** crossover control, meiotic recombination, crossover homeostasis, DSB formation, synaptonemal complex, Msh4-Msh5

## Abstract

Meiotic crossing over is essential for the segregation of homologous chromosomes. The formation and distribution of meiotic crossovers (COs), which are initiated by the formation of double-strand break (DSB), are tightly regulated to ensure at least one CO per bivalent. One type of CO control, CO homeostasis, maintains a consistent level of COs despite fluctuations in DSB numbers. Here, we analyzed the localization of proteins involved in meiotic recombination in budding yeast *xrs2* hypomorphic mutants which show different levels of DSBs. The number of cytological foci with recombinases, Rad51 and Dmc1, which mark single-stranded DNAs at DSB sites is proportional to the DSB numbers. Among the pro-CO factor, ZMM/SIC proteins, the focus number of Zip3, Mer3, or Spo22/Zip4, was linearly proportional to reduced DSBs in the *xrs2* mutant. In contrast, foci of Msh5, a component of the MutSγ complex, showed a non-linear response to reduced DSBs. We also confirmed the homeostatic response of COs by genetic analysis of meiotic recombination in the *xrs2* mutants and found a chromosome-specific homeostatic response of COs. Our study suggests that the homeostatic response of the Msh5 assembly to reduced DSBs was genetically distinct from that of the Zip3 assembly for CO control.

## Introduction

Meiotic recombination generates both crossovers (COs) and non-crossovers (NCOs). Crossing over during meiosis is essential to establish a chiasma as a physical connection between homologous chromosomes to ensure proper segregation of these chromosomes during the first meiotic division, meiosis I. Spo11 generates DNA double-strand breaks (DSBs) to initiate the recombination (Bergerat et al., 1997; Keeney et al., 1997). Spo11 forms a topoisomerase VI-like complex with Rec102, Rec104, and Ski8 (Robert et al., 2016; Claeys Bouuaert et al., 2021) and associates with two complexes, the Rec114-Mei4-Mer2 (RMM) and Mre11-Rad50-Xrs2 (MRX) complexes (Kee et al., 2004; Maleki et al., 2007). The number of DSBs exceeds the number of COs in budding yeast and other organisms; e.g., ∼90 COs from ∼170 DSBs in the budding yeast (Moens et al., 2002; Pan et al., 2011).

Meiotic CO formation is strictly regulated by several distinct mechanisms, which together are known as crossover control. Crossover interference negatively regulates CO formation to ensure even spacing and to limit the number of COs on each chromosome (Muller, 1916). Crossover assurance (or obligate CO) is a positive regulatory mechanism that ensures at least one CO on each homolog pair (Jones, 1984). It is thought that a balance between CO interference and assurance is the key feature of CO formation (Kleckner, 2006; Shinohara et al., 2015; Wang et al., 2019a). A third control mechanism, called CO homeostasis, was proposed based on studies of *spo11* hypomorphic mutants with differential DSB activities (Martini et al., 2006). CO homeostasis maintains a consistent number of CO events despite fluctuations in the number of meiotic DSBs (Martini et al., 2006). CO homeostasis may be a reflection of CO assurance mechanisms. However, the molecular mechanisms underlying CO homeostasis remain unknown. Moreover, the additional layer of CO control per nucleus basis, called CO covariation, is proposed (Wang et al., 2019b).

Meiosis-specific ZMM (Zip, Mer, Msh) or SIC (Synaptic Initiation Complex) proteins are components of recombination nodules on the synaptonemal complex (SC) and are required for CO formation and CO control; both CO interference and assurance (Sym et al., 1993; Hollingsworth et al., 1995; Nakagawa and Ogawa, 1999; Agarwal and Roeder, 2000; Novak et al., 2001; Tsubouchi et al., 2006; Shinohara et al., 2008). ZMMs include Zip1, Zip2, Zip3, Spo22 (also called Zip4), Mer3, Msh4, Msh5, and Spo16. Mer3 encodes a 5′-3′ DNA helicase and binds recombination intermediates (Nakagawa et al., 2001). Msh4 and Msh5 are homologs of *Escherichia coli* MutS, forming the Msh4-Msh5 complex (MutSγ), which binds to a recombination intermediate (Hollingsworth et al., 1995; Snowden et al., 2004). Msh4-Msh5 complex activates a nuclease activity of the Mlh1-Mlh3 complex (MutLγ) (Cannavo et al., 2020; Kulkarni et al., 2020; Dai et al., 2021). Zip2, Spo22/Zip4, and Spo16 form a complex (ZZS) required for SC elongation, which also binds to a recombination intermediate (Shinohara et al., 2008; De Muyt et al., 2018; Arora and Corbett, 2019). Msh4-Msh5 and ZZS complexes display differential roles in CO formation and control (Shinohara et al., 2008).

Coordinated activities of two recombinases, Rad51 and Dmc1, are required for proper strand invasion to form a displacement D-loop with a single-stranded DNA of the DSBs with homologous duplex DNA (Bishop et al., 1992; Shinohara et al., 1992; Shinohara et al., 2000; Shinohara et al., 2003). Stabilization of the D-loop to form a single-end invasion (SEI) or ejection of the invading strand is a critical regulatory step in the CO/NCO decision (Allers and Lichten, 2001; Hunter and Kleckner, 2001; Borner et al., 2004). The SEI is a specific intermediate for crossing over, which is converted into double Holliday junctions (dHJ) intermediate (Schwacha and Kleckner, 1994; 1995). Msh4-Msh5 complex stabilizes nascent joint molecules and activate a nuclease activity of the Mlh1-Mlh3 complex (MutLγ) for the resolution of dHJs into COs (Snowden et al., 2004; Cannavo et al., 2020; Kulkarni et al., 2020). Crossover interference is proposed to implement around the SEI formation (Kleckner, 2006; Shinohara et al., 2008). Moreover, recruitment of the Msh4-Msh5 complex to meiotic chromosomes depends on Zip3, but not other ZMM such as Zip2, Spo22/Zip4, or Mer3 (Shinohara et al., 2008). Zip3 has a conserved RING-finger motif and is predicted to function as Ubiquitin-E3 ligase or small ubiquitin-like modifier (SUMO)-E3 ligase (Perry et al., 2005; Cheng et al., 2006; Shinohara et al., 2008).

Xrs2 is a regulatory subunit of the MRX complex, which is required for DSB end resection, the DNA damage response, and nonhomologous end-joining during the vegetative cell growth (Johzuka and Ogawa, 1995; Tsubouchi and Ogawa, 1998; Usui et al., 1998; Palmbos et al., 2005; Matsuzaki et al., 2008; Mimitou and Symington, 2009; Ho and Burgess, 2011). In meiotic prophase I, Xrs2 is necessary for not only DSB end resection but also DSB formation, which could be mediated by the interaction with Mer2 (Arora et al., 2004). In addition, Xrs2 interacts with a meiosis-specific protein Pch2 and the interaction is involved in checkpoint signaling for meiotic recombination (Ho and Burgess, 2011). We previously isolated several *xrs2* mutations, and some showed defects in nonhomologous end-joining through interaction with DNA ligase IV in budding yeast (Shima et al., 2005). The mutants also had differential effects on the frequencies of meiotic DSBs, as seen with *spo11* hypomorphic mutants (Henderson and Keeney, 2004). The effects of various *xrs2* mutations on meiotic DSB frequencies could be explained by varied instability of mutant Xrs2 mutant proteins associated with these alleles (Shima et al., 2005).

Here, we used *xrs2* hypomorphic mutants to examine the relationship of global meiotic DSB frequencies with ZMM/SIC assembly on meiotic chromosomes as well as CO formation and control. Immuno-staining revealed that number of foci containing not only Rad51 and Dmc1 but also most ZMM proteins including Zip3 is proportional to DSB frequencies in the *xrs2* mutants. On the other hand, Msh5 ensembles on chromosomes showed a non-linear response to reduced DSB numbers. Our genetics analysis also confirmed CO homeostasis in response to reduced DSBs and showed a chromosome-specific effect of CO homeostasis. These suggest an important role of yeast MutSγ complex in the implementation of CO homeostasis, thus CO control.

## Materials and methods

### Strains and media

All yeast strains and their genotypes are shown in Supplementary Table S1. We used the isogenic *Saccharomyces cerevisiae* SK1 strain. The *spo11* mutant strains were derived from crossing a wild-type strain (MSY831) with SKY330 (*spo11-HA*) or SKY531 (*spo11-YF*), gifts from Dr. Scott Keeney. Synthetic complete media with 7.25 µM CuSO_4_ was used for *cup2* selection.

### Antibodies

Antibodies specific for Zip1 (generated in rabbit and rat), Zip3 (rabbit and rat), Mer3 (rabbit), Spo22 (chicken), Msh5 (rabbit), Dmc1 (rabbit), and Rad51 (rabbit and guinea pig) were described previously (Shinohara et al., 2008; Zhu et al., 2010; Matsuzaki et al., 2012; Sasanuma et al., 2013). We used two different rabbit anti-Msh5 antisera (Shinohara et al., 2008) and were able to observe two kinds of Msh5 foci dependent on a lot of Msh5 antibodies. In this study, we used an antibody that recognizes brighter ones specifically, which were used in our previous Chromatin-Immunoprecipitation of Msh5 (Nandanan et al., 2021). This might be a reason why we observed fewer Msh5 foci than in our previous report (Nishant et al., 2010).

### Cytology

Immunostaining of yeast meiotic chromosome spreads was performed as described (Shinohara et al., 2000). Stained samples were observed using an epifluorescence microscope (Zeiss Axioskop 2) and a ×100 objective (Zeiss AxioPlan, NA1.4). Images were captured with a CCD camera (Retiga; Qimaging) and processed using IP lab (Silicon) and Photoshop (Adobe). To count protein foci, >100 nuclei were counted for each sample. Pairs of foci were considered to colocalize if >50% of one side overlapped as described (Shinohara et al., 2000). The fluorescent intensity of Zip3 single-focus was measured by using the auto-thresholding signal intensity in Imaris software (Oxford Instrument). Strains used for this analysis were wild-type (NKY1551), *xrs2–314M* (MSY1992), *xrs2–228M* (MSY1524), and *xrs2–84M* (MSY1494).

### Genetic analysis of meiotic recombination

Genetics distances between markers and CO interference were analyzed using the MacTetrad 6.9.1 program (merlot.wekj.jhu.edu) as described (Shinohara et al., 2003; Shinohara et al., 2008; Shinohara et al., 2019). Parental haploid strains were mated for 3 h on YPAD (1% bacto-yeast extract, 2% bacto-peptone, 2% glucose, 0.004% adenine sulfate) plates at 30°C and then transferred onto SPM (0.3% potassium acetate, 0.02% raffinose) plates. After incubation at 30°C for 48 h, tetrads were dissected onto YPAD plates and incubated for 2 days. Genotyping was performed as described (Shinohara et al., 2003). To avoid aberrant clones (e.g., those containing mitotic COs), at least four independent crosses were carried out and pooled for further analysis. When analyzing interference or calculating genetic distances, we excluded tetrads with non-Mendelian segregation of a diagnostic marker from the analysis. Map distances were determined using Perkins equation: [distance in (cM)] = 100/2 (TT + 6NPD)/(PD + TT + NPD) (Perkins, 1949), where tetra types (TT), non-parental ditypes (NPD), parental ditypes (PD) observed. Standard errors were calculated using the Stahl Lab online tool (https://elizabethhousworth.com/StahlLabOnlineTools/). Interference values are expressed as the NPD ratio. The fraction of tetrads expected to be NPDs was determined from the Papazian equation: NPDexp = 1/2 [1 − TT − (1 − 3TT/2)^2/3^] (Papazian, 1952). To measure coincident double COs in adjacent intervals, the frequencies of tetrads with recombination in each of the two intervals were determined by summing TT and NPD tetrads for those intervals and dividing by the total number of tetrads (Shinohara et al., 2003). The expected frequency of coincident recombination is given by the product of two single-interval frequencies. Coefficient of coincidence (CoC) CO is calculated as follows: CoC = [CO(A∩B)]/[CO(A) × CO(B)], where A and B are CO frequencies in an adjacent single interval. Strains used for this analysis are wild-type (MSY4304/4245), *xrs2–314M* (MSY4314/4316), *xrs2–228M* (MSY4310/4312), and *xrs2–84M* (MSY4306/4308).

## Results

### The *xrs2* hypomorphic mutants showed differential DSB frequencies

**FIGURE 1:**
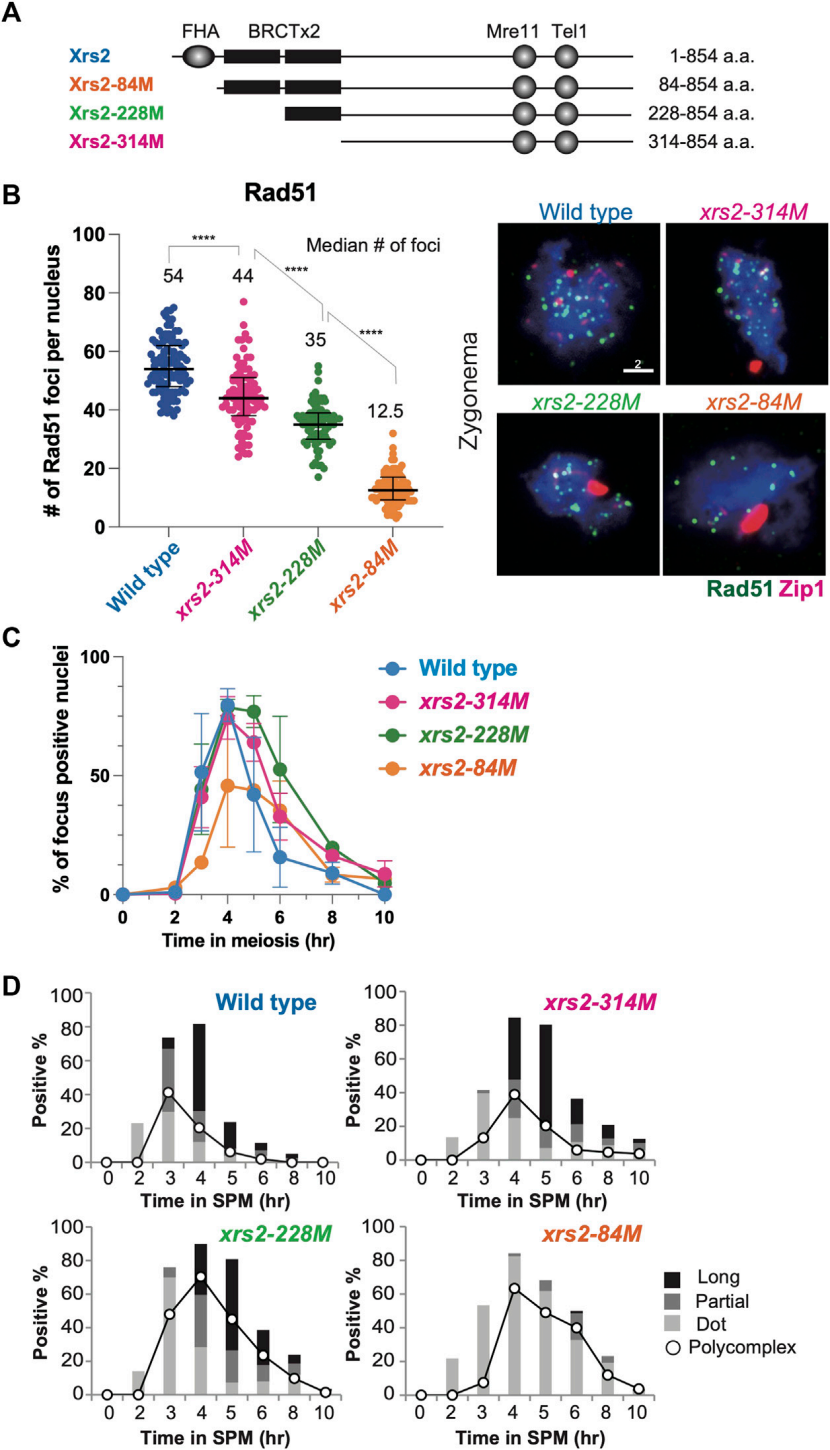
Rad51 focus formation and SC elongation in *xrs2* hypomorphic alleles. **(A)** Schematic representation of protein domain structure for yeast Xrs2 and truncated proteins encoded by *xrs2* hypomorphic alleles. The FHA domain and BRCA1 C-terminus (BRCT) domains, Mre11-binding and Tel1-binding domains are shown. **(B)** The number of Rad51 foci in each nucleus of wild-type, *xrs2–314M*, *xrs2–228M*, and *xrs2–84M* strains; wild-type (NKY1551), *xrs2–314M* (MSY 1992), *xrs2–228M* (MSY1524), *xrs2–84M* (MSY1494) was counted at the time point when the presence of focus positive nuclei in each strain peaked (4 h in wild type, *xrs2–314M*, and *xrs2–228M*, and 6 h in *xrs2–84M*). Median numbers of Rad51 were indicated. Error bar shows median and interquartile. Statistical significance was determined using Mann-Whitney *U*-test (****p < 0.0001). The right panel shows images of nuclear spreads in the zygotene stage that were labeled for Rad51 (green) and Zip1 (red). Scale bar = 2 µm. **(C)** Kinetics of Rad51-focus assembly and disassembly on meiotic nuclear spreads. A spread with more than 5 Rad51 foci was classified as a focus-positive nucleus. At each time point, more than 100 nuclei were counted. **(D)** Zip1-positive nuclei were classified into three categories: punctate foci (Dot, light gray), partial linear (Partial, gray), and full SC (Long, black). The kinetics of Zip1 poly-complex formation is represented by opened circles.

We previously reported that N-terminal truncations of Xrs2 significantly reduce meiotic DSB formation at the *HIS4–LEU2* hotspot (Shima et al., 2005). The *xrs2–84M*, *xrs2–228M*, and *xrs2–314M* mutants lack N-terminal 83, 227, and 313 amino acids, respectively (Figure 1A). On the other hand, even in the largest deletion, the *xrs2–314M* mutation does not cause any reduction of meiotic DSBs at the locus (Shima et al., 2005) with normal spore viability (Table 1). Despite the DSB reduction, *xrs2–228M* exhibits normal levels of spore viability. In contrast, the *xrs2–84M* allele, even though it encodes the smallest truncation (Figure 1A), shows significant reductions in spore viability of 52.4% (Table 1), as shown previously (Shima et al., 2005). The reduced spore viability in *xrs2–84M* cells is not caused by the deletion of the Forkhead-associated (FHA) domain of Xrs2 *per se* but rather by reduced levels of Xrs2 protein, as overexpression of Xrs2–84M protein rescues spore viability of the *xrs2-84M* mutant in a dose-dependent manner (Shima et al., 2005).

**TABLE 1:** Spore viability of the *xrs2* mutants.

Strain	Viable spores per ascus					Viability ±S.D.^a^
	4	3	2	1	0	
Wild type	1,200	69	27	4	8	96.8% ± 2.3%
*xrs2–314M*	1,200	61	25	2	6	97.3% ± 2.1%
*xrs2–228M*	1,367	282	100	11	34	90.9% ± 4.2%
*xrs2–84M*	1,275	590	759	215	1,269	52.4% ± 8.7%

^a^ Standard deviation of spore viability among independent crosses.

We further characterized meiotic defects for the three *xrs2* hypomorph mutants in more detail. We estimated the total number of meiotic DSBs in *xrs2* mutants by analyzing the number of immuno-stained Rad51 foci on meiotic chromosome spreads, which correspond to DSB sites (Bishop et al., 1992; Shinohara et al., 2000). We first counted the number of Rad51 foci in *spo11* hypomorphic mutants; *spo11-HA*/*spo11-HA*, *spo11-HA*/*spo11-Y135F* and *spo11-Y135F*/*spo11-Y135F*, which decreases DSB levels on chromosomes III, VII and VIII to ∼80%, ∼30% and 0%, respectively (Martini et al., 2006). The average Rad51-focus number in the wild type was 54.2 ± 0.7 (± Standard deviation [SD] at 4 h). The number at 4 h in the *spo11-HA*/*spo11-HA* and *spo11-HA*/*spo11-Y135F* was 39.4 ± 5.9 and 19.0 ± 0.1, respectively, while the *spo11-Y135F*/*spo11-Y135F* mutant formed little Rad51 foci as described previously (Bishop, 1994). The number of Rad51 foci is roughly proportional to DSB frequency on the three chromosomes in the various *spo11* hypomorphic mutants (Martini et al., 2006) (Supplementary Figure S1A). Rad51-focus number per spread could be used as a proxy for a total DSB number in a single nucleus.

We then studied the Rad51-focus number in the *xrs2* mutants and found that the average number of Rad51 foci within meiotic nuclei of wild type, *xrs2–314M*, *xrs2–228M*, and *xrs2–84M* was 54.2 ± 0.7, 42.8 ± 7.9, 35.7 ± 1.7 and 14.3 ± 4.8, respectively (Figure 1B). To avoid the kinetic effect, we analyzed the Rad51-focus number at 4, 5, or, 6 h, and then we decided to analyze 4-h samples which are when the peak of focus formation in each *xrs2* mutant (Figure 1C). Thus, from a relative decrease of Rad51 foci, we estimated that DSBs in *xrs2–314M*, *xrs2–228M*, and *xrs2–84M* mutants were reduced by 21%, 35%, and 74% compared with wild type, respectively. A similar reduction was observed for Dmc1 foci; an average number at 4 h of wild type, *xrs2–314M*, *xrs2–228M*, and *xrs2–84M* was 57.4 ± 3.3, 47.4 ± 5.6, 39.3 ± 1.9 and 18.3 ± 5.6, respectively. The *xrs2* mutant cells also showed slight delays in the disappearance of Rad51-focus positive spreads during meiosis (Figure 1C). The delayed disassembly of Rad51 foci suggests the role of the Xrs2 in meiotic DSB repair.

### Substantial DSB levels are required for Zip1 elongation

A meiosis-specific chromosome structure, the synaptonemal complex (SC), is formed between homologous chromosome axes. SC formation depends on meiotic recombination, thus DSB formation (Alani et al., 1990; Padmore et al., 1991). We also checked the effect of differential DSB levels in the *xrs2* mutants on SC formation by immune-staining analysis of Zip1 protein, which is a component of the central region of the SC (Sym et al., 1993). The Zip1-staining was classified into long, short lines, and dots (Figure 1D) as described previously (Shinohara et al., 2003). Like the wild type, fully-elongated Zip1 lines were observed in both the *xrs2–314M* and *xrs2–228M* mutants although the mutants showed only a 1-h delay in the appearance of long Zip1 lines as compared to the wild type, which is associated with a higher frequency of nuclei containing Zip1 poly-complex structures, an indicator for a defect in Zip1 elongation (Sym and Roeder, 1995). And the mutants delayed disassembly of Zip1 structure, consistent with delayed DSB repair in the mutants. The *xrs2–84M* mutant, which had the lowest level of DSBs (∼25%), showed a clear defect in Zip1 elongation with very few Zip1 long lines (Figure 1D). This indicated that substantial levels of DSBs were required for proper Zip1 elongation, thus chromosome synapsis. Similar results are seen with *spo11* mutants (Henderson and Keeney, 2004) and other mutants which reduced DSB levels (Bani Ismail et al., 2014).

The two BRCT-like domains of Xrs2 (amino acids 124-313; Figure 1A) have functions related to Pch2 (Ho and Burgess, 2011), which is required for normal SC formation and timely meiotic recombination progression (San-Segundo and Roeder, 1999; Borner et al., 2008). The *pch2* mutant cells show unusual localization of Hop1 protein on pachytene chromosomes with a delay in meiotic recombination (Borner et al., 2008). However, like in the wild type, we found dotty staining of Hop1 along long Zip1 lines in the *xrs2-314M* cells, which is different from long Hop1 lines on Zip1 lines seen in *pch2* cells (Supplementary Figure S2). The BRCT domains of Xrs2 do not appear to play a role in the Pch2 function in the Hop1 loading and/or unloading.

### Reduced DSBs decrease the association of ZMM/SIC and recombination proteins on meiotic chromosomes in *xrs2* mutants

Previously, it is shown that Zip3-GFP foci show a homeostatic response when DSBs are reduced in *spo11* hypomorphic mutants (Henderson and Keeney, 2004). First, we confirmed that Zip3 foci show the non-linear response in the *spo11* hypomorphic mutants by using our anti-Zip3 antibody without any tag-conjugation to Zip3 protein like previously reported (Supplementary Figures S1B, C). A steady-state number of Zip3 population was 61.5 (median), 58, 22, and 13 in wild-type, *spo11-HA*/*spo11-HA*, *spo11-HA*/*spo11-Y135F,* and *spo11-Y135F*/*spo11-Y135F*, respectively. The *spo11-HA*/*spo11-HA* mutant with ∼78% DSB level maintains a similar Zip3 focus number to the wild type (94%), indicating a non-linear relationship as shown previously (Martini et al., 2006). The *spo11-HA*/*spo11-Y135F* mutant with ∼29% DSB levels shows a higher Zip3 focus number (∼36% of the wild-type) that expected.

**FIGURE 2:**
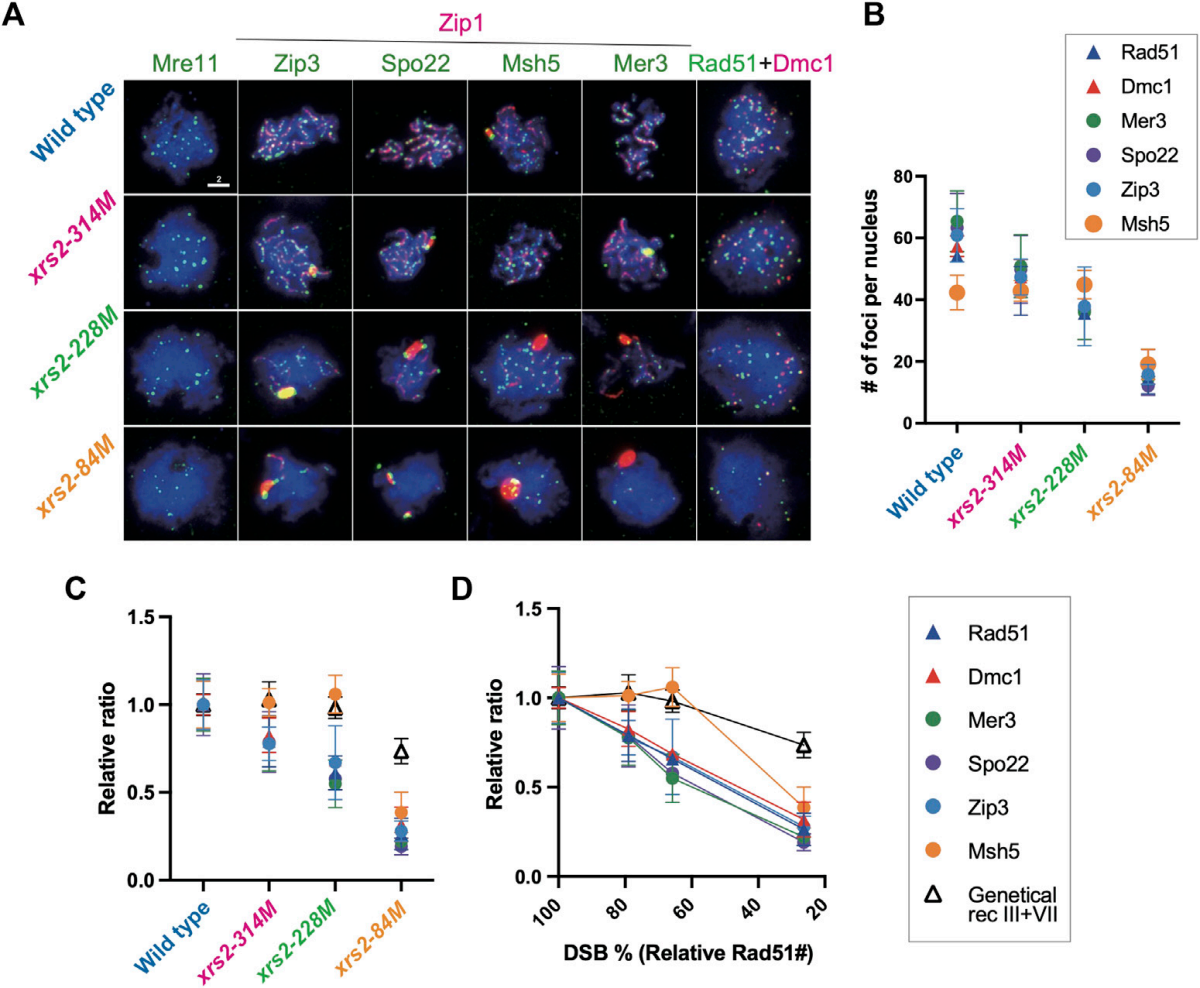
Assembly of recombination and ZMM/SIC components when DSB levels are reduced. **(A)** Colocalization of Zip1 (red; rat) and Zip3 (green; rabbit), Spo22/Zip4 (green; chicken), Msh5 (green; rabbit), or Mer3 (green; rabbit). Colocalization of Rad51 (green; guinea pig) and Dmc1 (red; rabbit). Genotypes are indicated. Wild-type (NKY1551), *xrs2–314M* (MSY1992), *xrs2–228M* (MSY1524), *xrs2–84M* (MSY1494) were used. Scale bar = 2 µm. **(B)** The number of foci of indicated proteins per nucleus in wild-type and *xrs2* mutants. The focus number in wild type, *xrs2–314M*, *xrs2–228M*, and *xrs2–84M* was counted at the time point when the presence of focus positive nuclei in each strain peaked (4, 5, or 6 h) as shown 1B. Error bars show the average and SD. **(C)** The number of foci of indicated proteins plotted against each average number of Rad51 foci (i.e., DSBs) associated with each strain (Figure 1B). Values are presented as a ratio relative to the wild type. Open triangles indicate relative CO frequencies as shown in **(B)**. Error bars show the average and SD. **(D)** The number of foci of indicated proteins in wild-type and *xrs2* mutants (non-normalized values). Error bars indicate the mean values and standard deviations from at least three independent experiments. Error bars show the average and SD. A black line with open triangles indicates relative CO frequencies of sums of analyzed intervals in chromosomes III and VII shown in Figure 4B. Values are presented as a ratio relative to the wild type.

We also analyzed the number of Zip3 foci as well as other ZMM foci including Spo22/Zip4, Msh5 and Mer3 when DSB frequencies are decreased by the *xrs2* hypomorphs (Figure 2A). Immunostaining was carried out and the focus number was counted at 4 h after meiosis entry for wild-type. To avoid the kinetic effect, we counted the focus number at 4 h (*xrs2–314M* and *–228M*) or 6 h (*xrs2–84M*) which is when the peak of focus formation in each *xrs2* mutant (Supplementary Figure S3A-representative kinetic analysis and Supplementary Figure S3D). The average number of foci per nucleus from four independent time courses (more than 100 focus-positive nuclei were analyzed for each counting) for Rad51, Dmc1, Zip3, and Msh5, and from two independent time courses for Spo22/Zip4 and Mer3 are shown in Figure 2B. As shown above (Figure 1), the average numbers of Rad51 and Dmc1 foci at 4 h in wild-type nuclei were 54.2 ± 0.7 (SD) and 57.4 ± 3.3, respectively (Figure 2B), which is consistent with a previous study (Shinohara et al., 2000). The ZMM/SIC proteins Zip3, Spo22/Zip4, and Mer3 exhibited similar numbers of foci in wild-type nuclei: 60.9 ± 8.6, 63.4 ± 11, and 65.5 ± 9.8, respectively (Figure 2B). There were few significant differences in a steady-state number of foci between ZMM foci with either Zip3, Mer3, or Spo22/Zip4, and the RecA-like recombinases (Figures 2A,B). Of note, the steady state number of Zip3 foci in the wild type detected by anti-Zip3 was almost the same as the numbers reported to Zip3-myc (∼60 foci) by two independent groups (Yoon et al., 2016; Hong et al., 2019; Tan et al., 2022) but about twice than that reported to Zip3-GFP (Henderson and Keeney, 2004).

When *xrs2* mutants were examined, the focus number of Rad51, Dmc1, and ZMM/SIC proteins such as Zip3, Spo22/Zip4, and Mer3 reduce linearly along with meiotic DSB frequencies in the mutants (Figures 2B–D). Like Rad51/Dmc1 foci, focus numbers of Zip3, Spo22/Zip4, and Mer3 are decreased when DSB frequencies are reduced. When compared with the number, Zip3, Spo22/Zip4 and Mer3 shows linear correlation with Rad51 (*R* = 0.999, 0.999, 0.994, and 0.982 for Dmc1, Zip3, Spo22/Zip4 and Mer3, respectively). These suggest that, like Rad51/Dmc1 recombinases, Zip3-, Spo22/Zip4-, and Mer3-focus number is linearly correlated with DSB number. Moreover, these are consistent with the result that the focus formation of these proteins depends on meiotic DSB formation (Agarwal and Roeder, 2000; Nakagawa et al., 2001; Shinohara et al., 2008).

While the Zip3-focus number shows a linear relationship with DSB frequency in the *xrs2* mutants (Figure 2B; see below), the number of Zip3-GFP foci (Henderson and Keeney, 2004) and Zip3 foci detected by anti-Zip3 (Supplementary Figure S1B) exhibit a non-linear relationship in *spo11* hypomorph mutants. This suggests a role of N-terminal regions such as the FHA domain and/or BRCT repeat in the homeostatic response of ZMM foci of Zip3 as well as Mer3 and Spo22/Zip4 to reduced DSBs.

### Msh5-focus numbers are maintained even with reduced meiotic DSBs are reduced

We found that Msh5 foci showed a unique behavior on the chromosomes among ZMM proteins. In the wild type, the average (steady-state) number of Msh5 foci is 42.4 ± 5.6, which is significantly lower than those of Rad51, Dmc1, Zip3, Mer3, and Spo22/Zip4 (Figures 2A, B), suggesting the presence of a regulatory mechanism for Msh5-focus formation.

Different from Zip3, Spo22/Zip4, and Mer3 as well as Rad51/Dmc1, Msh5 foci showed a non-linear relationship in its number to reduced DSBs in the *xrs2* mutants. The number of Msh5 foci in the *xrs2–314M* and *xrs2–228M* strains was 42.9 ± 3.4 and 44.9 ± 4.6, respectively, which is similar to that in the wild type of 42.4 (Figure 2B; Supplementary Figure S3B). Thus, Msh5 foci exhibited homeostasis as DSBs were reduced by ∼40% (in *xrs2–228M*). This non-linear response of ZMM foci was reported to the foci containing Zip3-GFP (Henderson and Keeney, 2004) and Zip3 foci detected by anti-Zip3 (Supplementary Figure S1B) in *spo11* hypomorph mutants. On the other hand, more dramatic reductions in meiotic DSBs did affect the Msh5-focus number, as the number of Msh5 foci in *xrs2–84M* mutant cells decreased substantially to 19.1 ± 4.9, which represented 38.6% of wild type. However, this reduction of Msh5-focus number in the *xrs2–84M* mutant is much milder than those of Rad51, Zip3, Spo22/Zip4, and Mer3 (26.4, 27.9, 19.2, 22.1%, respectively, in Figures 2C, D). This suggests that the homeostatic response of Msh5 foci substantially operates even in the *xrs2–84M* mutant.

Notably, the similar non-linear relationship was seen for Msh5 foci in *spo11* hypomorphic mutants (Supplementary Figure S1C). Importantly, the number of Msh5 foci (41 [median] and 39 foci in wild-type and *spo11-HA*/*spo11-HA* strains, respectively) was lower than that of Zip3 foci (61.5 and 58 in wild-type and *spo11-HA*/*spo11-HA* strains, respectively, in Supplementary Figure S1C), supporting a distinct response between Msh5 and Zip3 foci.

**FIGURE 3:**
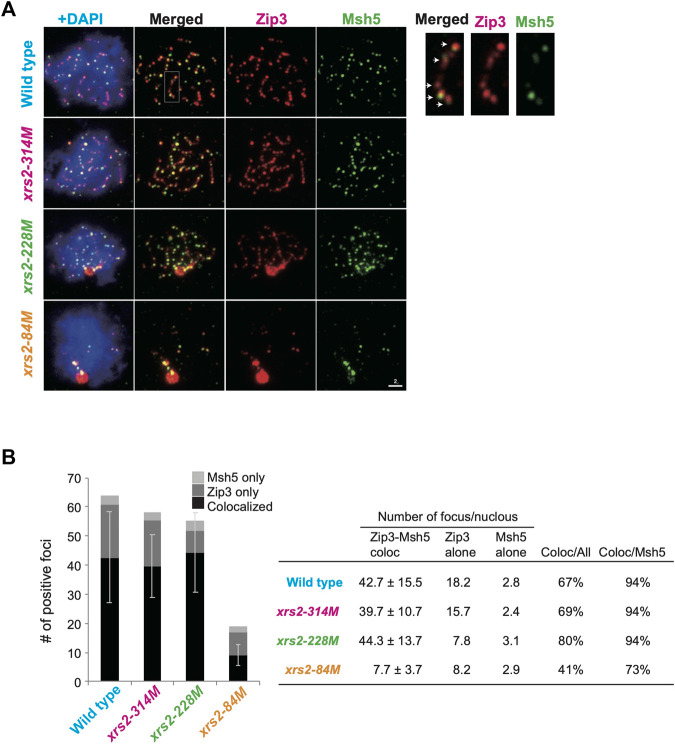
Colocalization of Zip3 and Msh5 on meiotic chromosomes **(A)** Meiotic nuclear spreads were stained for Zip3 (red) and Msh5 (green) by using anti-Zip3 (red; rat) and anti-Msh5 (green; rabbit). Anti-Zip3 used here was different from that in Figure 2. Genotypes are indicated. A magnified image of a wild-type sample is shown on the right. Arrows show colocalization of Zip3 and Msh5. Scale bar = 2 µm. **(B)** Colocalization frequencies for Zip3 and Msh5. Foci were classified into three categories: Zip3 and Msh5 (colocalized, dark gray), Zip3-only (pale gray), and Msh5-only (gray). The average numbers with standard deviations of foci in three categories in wild-type and *xrs2* mutants are shown. The number of nuclei analyzed in wild type (4 h), *xrs2-314M* (4 h), *xrs2-228M* (4 h), and *xrs2-84M* (5 h) is 102, 105, 105, and 59, respectively.

Msh5-focus formation depends on Zip3 (and Zip1), but not on Spo22/Zip4 or Spo16 (Shinohara et al., 2008). We analyzed the relationship between the Zip3 and Msh5 localization by double staining of “pachytene” cells (at 4 h in wild-type and 5 h in *xrs2* mutants) (Figure 3A). Medians of Zip3 foci number distribution in wild type, *xrs2–314M*, *xrs2*–*228M,* and *xrs2–84M* cells were 63, 56, 54, and 16, respectively (Supplementary Figure S3C). Although the number of Zip3 foci co-stained with Msh5 in the *xrs2–84M* is similar to that co-stained with Zip1 shown in Figure 2B (16 versus 14), the focus number of Zip3 co-stained with Msh5 in the *xrs2–314M* and *xrs2*–*228M* mutants were significantly higher than that co-stained with Zip1 (56 versus 45 and 54 versus 35 in the *xrs2–314M*, and *xrs2*–*228M* mutants). The focus-number distribution indicates variations of the focus number are smaller in the double-staining of Msh5 and Zip3 than in the co-staining with Zip1 (Supplementary Figures S3B, C). This suggests that Zip3 co-stained with Msh5 showed a homeostatic response as shown previously (Henderson and Keeney, 2004) and in this study (Supplementary Figure S1C). On the other hand, in this double staining of Zip3 and Msh5, the medians of Zip3 focus number distribution were 47, 43, 49, and 10 in wild-type, *xrs2–314M*, *xrs2–228M,* and *xrs2–84M* mutant cells, respectively (Supplementary Figure S3C), which are not different from those co-staining with Zip1. A simple interpretation is a kinetic effect such that the focus numbers of ZMM proteins in the pachytene stage are more than those in earlier stages. Supporting this idea, the focus numbers were increased in later time points of prophase I, especially of Zip3 foci in the *xrs2-228M* mutant (Supplementary Figure S3A).

Importantly, even in the double-staining of Msh5 and Zip3, the Zip3-focus number is higher than the Msh5-focus number in any strains (Figure 3B). In the wild type, 67% of Zip3 foci colocalized with Msh5, and 94% of Msh5 foci colocalized with Zip3. In *xrs2* mutants, Zip3-Msh5 colocalization frequencies in Zip3 foci were 69%, 80%, and 41% for the *xrs2–314M*, *xrs2–228M,* and *xrs2–84M* mutants, respectively. In addition, 94%, 94%, and 73% of Msh5 foci colocalized with Zip3 in *xrs2–314M*, *xrs2–228M,* and *xrs2–84M* mutants, respectively (Figure 3B). This is consistent with the idea that some Zip3 foci become a site for Msh5 assembly, which is regulated by DSB levels. In addition, Zip3 foci colocalized with Msh5 seemed brighter than Zip3 without Msh5 (Figure 3A; Supplementary Figure S1E). We speculate the presence of stepwise homeostatic response of ZMM-focus assembly in response to meiotic DSBs in a context-dependent manner (See Discussion).

### CO homeostasis functions more effectively on chromosome VII

**FIGURE 4:**
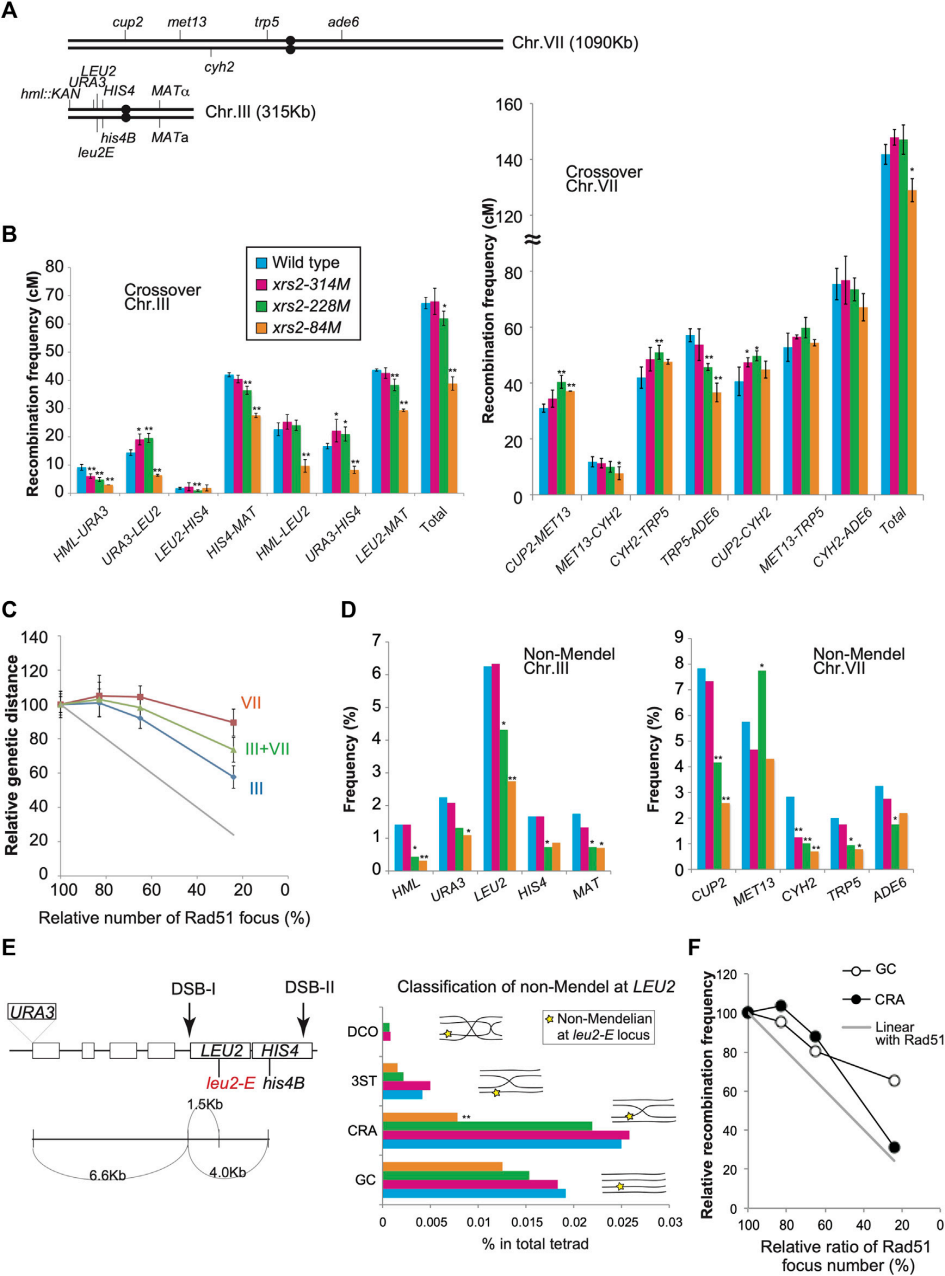
Genetic analysis of *xrs2* hypomorphic alleles. **(A)** Schematic representation of genetic markers on chromosomes VII and III. **(B)** CO frequencies within indicated genetic intervals on chromosomes III and VII. Genotypes are color-coded. Error bars indicate the standard deviation from four independent crosses. Wild-type (MSY4304/4245), *xrs2–314M* (MSY4314/4316), *xrs2–228M* (MSY4310/4312), and *xrs2–84M* (MSY4306/4308) were used. Statistical significances were calculated by using Student’s t-test. **(C)** Relationships between the CO frequencies and DSB levels. The *x*-axis values indicate the mean number of Rad51 foci for each *xrs2* mutant relative to that in the wild type. The *y*-axis values indicate the mean genetic distance sums for each *xrs2* mutant relative to the wild type (for chromosomes III or VII). The gray line shows a linear relationship. **(D)** Non-Mendelian segregation frequencies at the indicated genetic loci are shown. Statistical differences were analyzed using Fisher’s exact test with Yates correction. **(E)** Schematic representation of the *HIS4*–*LEU2* hotspot on chromosome III. Locations of the *leu2-E* and *his4B* mutations are shown. Rectangles represent genes. The non-Mendelian fraction at the *LEU2* locus was classified by analyzing the linkage of the *URA3*, *LEU2*, and *HIS4* loci. GC; gene conversion at the *LEU2* locus without CO between *URA3* and *HIS4*; CRA; CO-associated gene conversion on the same strand as the CO, 3ST; CO-associated gene conversion on the strand lacking CO, DCO; gene conversion associated with a double CO. **(F)** Non-linear relationship of COs or NCOs derived from the non-Mendelian fraction at the *leu2-E*/*LEU2* heteroalleles. The *x*-axis values indicate the relative (the mean) numbers of Rad51 foci for each *xrs2* mutant relative to the wild type. The *y*-axis values indicate the relative frequencies of COs or NCOs for each *xrs2* mutant. The gray line shows a linear relationship. Asterisks indicate statistically significant differences between the *xrs2* mutant and wild type (**p < 0.01, *p < 0.05) (Supplementary Table S4).

**FIGURE 5:**
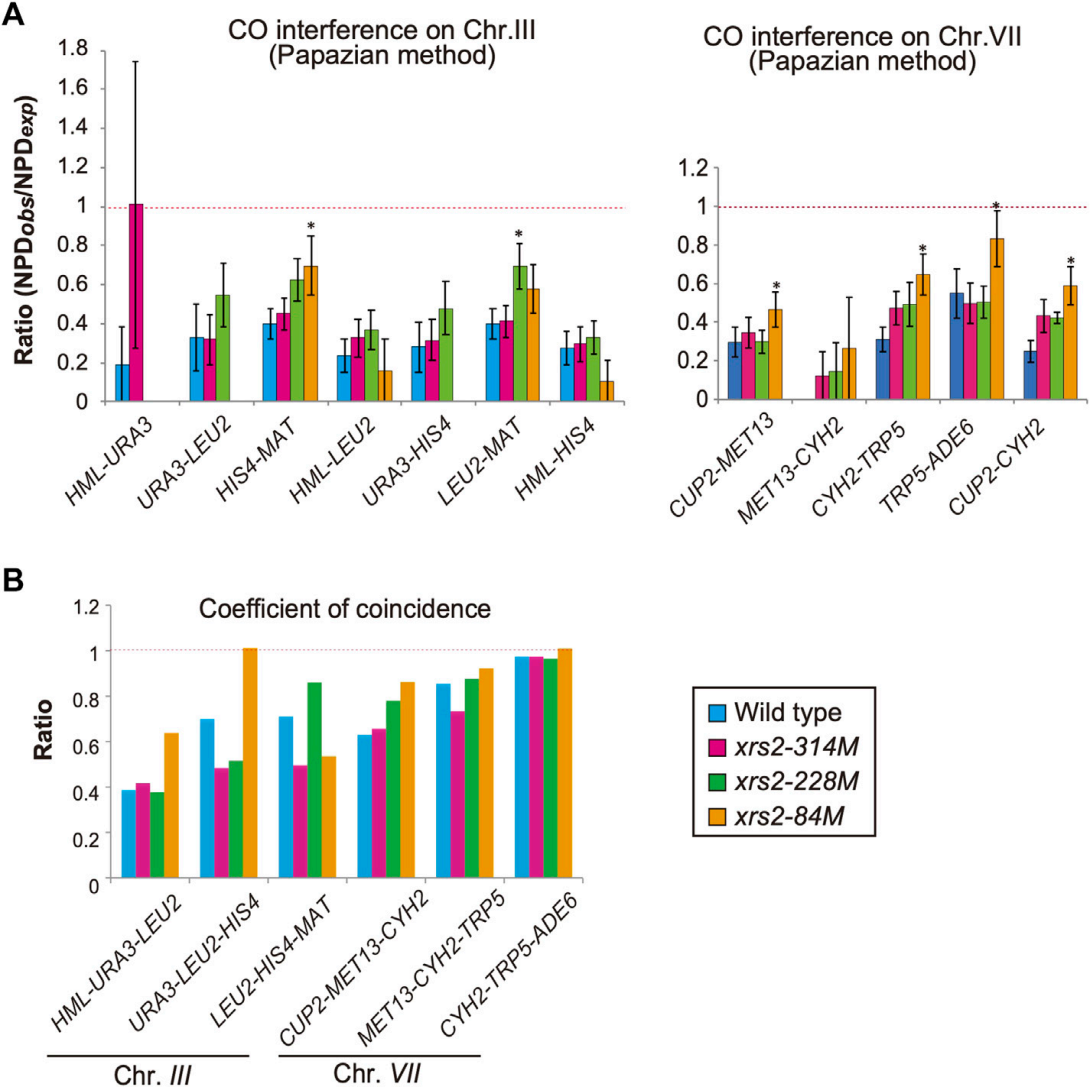
CO interference in *xrs2* mutants. **(A)** CO interference for indicated genetic intervals on chromosomes III and VII. Genotypes are color coded. The NPD_obs_/NPD_exp_ ratio for three intervals was calculated from TT and PD (Supplementary Tables S2, S3). A ratio of 1 indicates no interference. A Ratio <1 indicates positive interference. Error bars indicate the standard error of NPD ratios and the statistical significance of the difference in NPD ratio between the wild type and each *xrs2* mutant was confirmed by an overlap of the SE value around the map distance or NPD ratio. (Supplementary Tables S2, S3). **(B)** The Coefficient of coincidence (CoC) of COs between adjacent intervals on chromosomes III and VII in wild-type and *xrs2* mutants are shown. A ratio of 1 indicates no interference. A ratio of <1 indicates positive interference.

Using *spo11* alleles with ∼80%, ∼70%, and ∼20% of wild-type DSB levels, Martini et al. (2006) showed CO homeostasis which maintains CO levels despite reduced meiotic DSBs. We, therefore, asked whether *xrs2* alleles with ∼80%, ∼65%, and ∼25% of wild-type DSB levels also exhibited CO homeostasis since Zip3 foci showed non-linear response to reduced DSBs in the *spo11*-mutants, but not in the *xrs2* mutants. We measured CO and NCO frequencies by the dissection of tetrads for SK1 yeast strains with different genetic markers on chromosomes III and VII; a short chromosome (chromosome III with a synthetic recombination hotspot at the *HIS4* locus [*HIS4*–*LEU2*]) and a long chromosome (chromosome VII) (Higashide and Shinohara, 2016) (Figure 4A). We analyzed the segregation of genetic markers associated with these chromosomes in >1,200 tetrads with four viable spores to calculate CO frequencies (in centimorgans; cM) for each strain (Supplementary Tables S2, S3); the number of tetrads analyzed was larger than that in the previous study (>750 four-viable tetrads; Martini et al., 2006). Tetrad analysis revealed that wild type of SK1 strain, *xrs2–314M*, *xrs2–228M*, and *xrs2–84M* strains had spore viabilities of 96.8%, 97.3%, 90.9%, and 52.4%, respectively (Table 1). The results of genetic analysis in wild-type controls (Figures 4, 5) are generally consistent with our previous report (Shima et al., 2005; Higashide and Shinohara, 2016; Shinohara et al., 2019). We assumed that DSB distribution in various *xrs2* strains is not altered and DSB levels are uniformly reduced along the genome, which is a simple but cautious assumption given that DSB formation was controlled in various ways (Yadav and Claeys Bouuaert, 2021) and DSBs are proceeded differentially in the mutant (see above).

Chromosome VII: The *xrs2–314M* (∼80% DSBs) and *xrs2–228M* (∼65%) mutants showed wild-type levels of total CO frequency between the *CUP2* and *ADE6* loci, 127.9 ± 14.6 (105%) and 127.1 ± 7.9 (104%) cM, respectively, compared with 121.8 ± 9.4 cM for wild type (Figure 4B; Supplementary Table S2). The *xrs2–84M* mutant (∼25% DSBs) slightly, but significantly reduced CO frequency with 109 ± 9.6 cM (89%) relative to the wild type. These showed that CO levels responded non-linearly to a reduction of DSB frequencies (Figure 4C). This supports the CO homeostasis in response to DSB reduction (Martini et al., 2006). Among different intervals inspected, we see the interval-specific response to reduced DSB levels. The *xrs2–314M* mutant (∼80% DSBs) showed similar CO frequencies in all intervals to the wild type. The *xrs2–228M* mutant (∼65%) showed a slight reduction in the *TRP5-ADE6* interval and similar CO levels in the *MET13-CYH2* interval compared to the wild type. Interestingly, the mutant showed significantly increased CO frequencies in two intervals (*CUP2-MET13* and *CYH2-TRP5*) relative to the wild type. This increased response of CO frequencies in response to DSB was not reported in the previous study (Martini et al., 2006). For *xrs2–84M*, two of the four single intervals showed wild-type levels of CO, despite a 76% reduction in meiotic DSBs. While the *CUP2-MET13* interval increased the frequency compared to the wild type, the *TRP5-ADE6* interval in the mutant significantly reduced CO frequency.

Chromosome III: The *xrs2–314M* mutant (∼80% DSB) showed wild-type levels of the total CO frequency between the *HML* and *MAT* loci, 68.0 ± 5.5 cM compared with 67.4 ± 3.1 cM for wild-type (Figure 4B; Supplementary Table S3). The *xrs2–228M* mutant (∼65%) slightly decreased CO frequency of 62.0 ± 4.0 cM (92%). On the other hand, the *xrs2–84M* mutant (∼25% DSBs) reduced 57.7% of the wild-type level (38.9 ± 4.4 cM), which is much higher than the expected frequency without the homeostasis (∼16.9 cM). For each interval, the *xrs2–314M* mutant slightly decreased CO in one interval (*HML-URA3*) among four intervals on the chromosome. The *xrs2–228M* mutant (∼65%) and the *xrs2–84M* mutant (∼25% DSBs) showed decreased CO frequencies in two and three intervals, respectively. In the *LEU2-HIS4,* the *xrs2–314M* and *xrs2–228M* mutants maintained wild-type CO levels while the *xrs2–84M* mutant decreased COs relative to the control (see below). Taken together, these suggested that chromosome III is less robust for CO homeostasis than chromosome VII (Figure 4C). Similar results were obtained in the previous study although it was not emphasized (Martini et al., 2006). However, we do need more caution on the interpretation of recombination on chromosome III, since our strains, but not a previous strain, contains an unusual recombination hot spot, *HIS4–LEU2* on the chromosome.

When the combined CO frequencies on chromosomes III and VII are compared with the total DSBs level in the *xrs2* mutants, the CO frequencies are maintained even in the *xrs2–228M* mutant with ∼65% DSBs (Figures 4B, C). This CO homeostasis is roughly correlated with that seen for Msh5 foci (Figure 2D).

### NCO formation is sensitive to reduced levels of meiotic DSBs

Next, we analyzed frequencies non-Mendelian segregation at 10 genetic loci on chromosome III and VII in different *xrs2* alleles (Figure 4A). For *xrs2–228M* (∼65% DSBs) mutant, four of five loci on chromosome III and four of five loci on chromosome VII showed significant decreases in non-Mendelian segregation frequencies (Figure 4D; Supplementary Table S4). The *xrs2–314M* mutant (∼80% DSBs) reduced the frequency only at the *CYH2* locus. The *xrs2–228M* mutant, which maintains CO frequencies with 65% DSB reduction, seems to show reduced NCO. Strangely, the *xrs2–228M* mutant increased the frequency at the *MET13* locus. For the *xrs2-84M* (∼25% DSBs), four of five loci on chromosome III and three of five loci on chromosome VII showed significant decreases in non-Mendelian segregation frequencies. The other three loci (*HIS4*, *MET13*, and *ADE6*) showed reduced frequencies relative to the wild type, but the difference is not significant. At the *LEU2*, *CUP2,* and *MET13* loci, frequencies in the *xrs2-84M* mutant are significantly lower than those in the *xrs2–228M* mutant. In most cases, however, except for the *HML* locus, reductions in non-Mendelian segregation were not proportional to reductions in meiotic DSBs, as reported (Martini et al., 2006) (Figure 4D; Supplementary Table S4).

*HIS4-LEU2*: Non-Mendelian segregation is thought to result from a simple gene conversion or mismatch repair of heteroduplexes formed during CO formation (Nicolas and Rossignol, 1983; White et al., 1985). The *URA3*–*LEU2*–*HIS4* interval on chromosome III has an artificial meiotic DSB hotspot (DSB-I) with *leu2E* mutation (an insertion allele of the *Eco*RI site) and *URA3* insertion (Figure 4E). As *leu2E* and the *URA3* insertion are very close to the DSB-I site (∼1.5 and ∼6.6 kb way, respectively), we assumed that *LEU2*/*leu2* gene conversion with or without flanking crossover would come from DSB-I. Non-Mendelian tetrads of *LEU2*/*leu2E* heteroalleles (3 Leu+: 1 Leu- or 1 Leu+: 3 Leu-segregation) were initially selected, and then sorted into four classes based on the linkage with flanking markers, *URA3* and/or *HIS4* alleles; GC, Gene conversion; CRA, Crossover associated gene conversion; DCO, double CO; three strands, 3ST, gene conversion associated with incidental CO (schematic figures in Figure 4E middle graph). A previous study showed 40% and 14% of wild-type levels of DSB-I in the *xrs2-228M* and *xrs2-84M* mutants, respectively (Shima et al., 2005). The *xrs2-228M* maintained ∼90% of wild-type CO level (CRA and DCO classes). Moreover, decreased level of COs (CRA and DCO) in the *xrs2-84M* mutant (∼30%) is much higher than reduced DSB levels (14%) at the locus. These support the idea that CO homeostasis is operating at this locus (Figure 4F), which was not seen in the physical analysis of this locus in the previous study (Martini et al., 2006). GC frequencies were also reduced in response to decreased DSB levels (Figure 4F), although higher than expected in the *xrs2-228M* (80% to expected 40%) and *xrs2-84M* mutants (65% to expected 14%). These high frequencies of meiotic recombination in the *xrs2-228M* mutant cannot be explained by DSB-I. These might come from an event at DSBs other than DSB-I such as DSB-II.

### Reduced levels of meiotic DSBs weaken CO interference

CO interference negatively regulates CO formation to maintain the appropriate number and spacing of COs (Muller, 1916). A previous study on CO interference in response to reduced DSBs (Martini et al., 2006) showed that the interference is maintained when DSB frequencies are reduced. To confirm this, we also analyzed CO interference in *xrs2* mutants using the same data described above. In each interval, the tetrads were classified into three classes with a different combination of flanking markers: parental ditypes (PD), tetra types (TT), and non-parental ditypes (NPD). NPD is a tetrad class with “double” COs involving four chromatids in an interval, whose an expected frequency, NPD_exp_, is calculated from a frequency of the TT class, which mainly contains a single CO event in the interval (Papazian, 1952). First, we used the Papazian method to examine the ability of a CO to interfere with coincident COs in the interval by determining the ratio of observed NPD (NPD_obs_) to NPD_exp_ (Figure 5A, Supplementary Tables S2, S3). In the wild type, the ratio of NPD_obs_ to NPD_exp_, called the NPD ratio, is indicative of interference when the ratio is <1. Indeed, as reported previously (Higashide and Shinohara, 2016; Shinohara et al., 2019), the NPD ratio of seven intervals on chromosome III and VII in the wild type is 0.19–0.55 (Figure 5A; Supplementary Tables S2, S3), confirming CO interference within these intervals. In contrast, we did not detect any NPD tetrads within the *MET13*-*CYH2* interval after analyzing >1,200 tetrads, indicating the presence of a strong interference in this interval (Figure 5A; Supplementary Tables S2, S3).

We then analyzed tetrads for the *xrs2* mutants. For all *xrs2* mutants, the NPD ratio associated with each interval on chromosomes III and VII was <1 (except for *HML–URA3*) (Figure 5A; Supplementary Tables S2, S3). In all cases, the ratio is statistically significant from one (no interference), showing that the CO interference is maintained in the mutants. Although the NPD ratio at the *HML–URA3* in the *xrs2–314M* was about 1; the number of NPD_obs_ in this interval was too low (1 for wild type, 2 for *xrs2–314M*, and 0 for the other alleles) to draw any significant conclusions. As Papazian’s NPD analysis requires an NPD fraction, which we could not obtain for some chromosome III intervals (e.g., *URA3-LEU2*) in *xrs2–84M* because of severe reductions of COs in the mutant. These suggest that CO interference could function even when the number of DSBs was reduced to 20% levels of the wild type. However, as discussed above, this idea depends on the similar DSB distribution along these reporter chromosomes in the *xrs2* mutants to that in the wild type.

When compared with the NPD ratios in various *xrs2* mutants with those in the wild type, we found that the NPD ratios in the *HIS4-MAT*, *CUP2-MET13*, *CYH2-TRP5*, and *TRP5-ADE6* intervals in the *xrs2–84M* mutant (∼20% DSBs of wild type) are significantly higher than corresponding ratio in wild-type cells (Figure 5A; Supplementary Tables S2, S3). Higher NPD ratios in the mutant relative to the wild type are also observed in the *HIS4-MAT* and *CYH2-TRP5* intervals of the *xrs2–228M* mutant (∼65% DSBs) as well as in the *CYH2-TRP5* intervals of the *xrs2–314M* mutant (∼80% DSBs). These suggest weakened CO interference when DSB frequencies are reduced by the *xrs2* mutations.

We also analyzed the frequency of double COs in two adjacent intervals using the tetrad data (above) for the coefficient of coincidence (CoC; Figure 5B; Supplementary Table S5). CoC is a ratio of an observed number of tetrads with simultaneous COs in adjacent intervals to an expected number of double crossovers, which is obtained from frequencies of a CO in each interval (Muller, 1916). In the wild type, five adjacent intervals showed a CoC ratio <1 (CoC for *CYH2–TRP5–ADE6* is less than one but not statistically significant). The *xrs2–314M* and *xrs2–228M* mutants exhibited CoC ratios that were <1 for the five intervals. The *xrs2–84M* mutant showed CO interference for four adjacent intervals, but in the *URA3–LEU2–HIS4* interval the CoC was 1.01 (Figure 5B; Supplementary Table S5). This indicated that a ∼80% reduction in meiotic DSBs caused a defect in CO interference at *HIS4–LEU2* hotspot on chromosome III, which is an abnormal response of the *xrs2–84M* mutant in CO and NCO formation (Figures 4E, F).

CoCs in the *xrs2* mutants were compared to those in the wild type. In the *xrs2–84M* mutant, CoC ratios are higher in four adjacent intervals, *HML-URA3-LEU2*, *URA3-LEU2-HIS4*, *CUP2-MET13*-*CYH2*, *MET13*-*CYH2-TRP5,* and *CYH2-TRP5-ADE6* while lower in one interval, *LEU2-HIS4-MAT*. The *xrs2–228M* mutant shows a higher CoC ratio in the *LEU2-HIS4-MAT* and *LEU2-HIS4-MAT*, but lower in the *URA3-LEU2-HIS4*. These support the idea that CO interference is weakened when DSB frequencies are largely decreased by the *xrs2* mutations.

## Discussion

Here we analyzed meiotic CO formation and the assembly of proteins involved in CO formation in *xrs2* hypomorphic mutants with different levels of DSB formation. The *xrs2–314M* and *xrs2–228M* mutants exhibited 20% and 35% reductions in meiotic DSBs, respectively, but wild-type levels of CO formation and spore viability. This indicated that CO homeostasis functions in the *xrs2* mutant cells, as it does in *spo11* mutants (Martini et al., 2006). In contrast, when DSBs were reduced by ∼80%, which was the case in *xrs2–84M* mutant, CO homeostasis weakened. We also described the homeostatic response of the formation of foci containing a ZMM protein, Msh5, but not Zip3 in the *xrs2* mutants. These suggest that CO homeostasis is mediated by Msh5, thus, Msh5-containing MutSγ complex with Msh4.

### Foci containing Msh5 exhibit homeostasis in response to reduced DSBs

By analyzing the number of foci containing different meiotic recombination proteins in an *xrs2* mutant with reduced meiotic DSB formation, we found a linear correlation of the number of ensembles containing ZMM/SIC proteins (Zip3, Mer3, and Spo22/Zip4, but not Msh5) as well as Rad51 and Dmc1 (Figure 2B). The steady-state number of these foci is similar among the proteins. If the lifespan of these foci is similar, we expect the same number of ensembles of these proteins. ZMM-focus formation is independent of Rad51/Dmc1-focus formation (Shinohara et al., 2008), although the formation of both Rad51/Dmc1 and ZMM foci requires the formation of ssDNAs at meiotic DSB sites. These suggested that ensembles containing Zip3, Mer3, and Spo22/Zip4 were closely associated with the ssDNA region near Rad51/Dmc1. This is consistent with recent biochemistry and genome-wide mapping of ZMM proteins including Zip3 which bind the DSB sites in addition to chromosome axes (Serrentino et al., 2013; De Muyt et al., 2018).

Although this study revealed the linear relationship of Zip3-focus number to DSB level in the *xrs2* mutants, a previous study showed a non-linear relationship between Zip3 foci and DSBs in *spo11* hypomorphic mutants (Henderson and Keeney, 2004). One critical difference between our study and this previous report was the antibody used to detect Zip3 foci. We used two independent raised polyclonal antibodies against recombinant Zip3 protein generated by our lab (see Materials and Methods) that detected 60.9 ± 8.6 (generated in rabbit) and 60.8 ± 14.5 (generated in rat) foci in wild-type zygotene/pachytene cells. This number is compatible with those reported for Zip3-myc (∼60 foci) by two independent groups (Yoon et al., 2016; Hong et al., 2019; Tan et al., 2022). On the other hand, the previous report detected only 35.3 ± 6.2 foci of a Zip3-GFP fusion protein on elongated SCs in wild-type (*ZIP3-GFP*) cells using an antibody against GFP. GFP-tagging of Zip3 may therefore affect the chromosomal localization of Zip3 proteins. Alternatively, an anti-GFP antibody could detect only the subfraction of Zip3 on chromosomes, which is resistant to reduced DSB levels. Indeed, by using our Zip3 antibody, we also found a non-linear response of the Zip3-focus number in the *spo11* hypomorph mutants. A steady-state number of Msh5 foci (∼40) is much lower than that of Zip3 and other recombination foci (∼60). Given Msh4/5-focus kinetics is similar to those of Rad51/Dmc1 (Zhu et al., 2021 and here), a difference in the life span could not explain the difference in the number of foci, suggesting the presence of a distinct regulatory mechanism to assembly Msh5-ensembles than those of Rad51 and other ZMM proteins.

**FIGURE 6:**
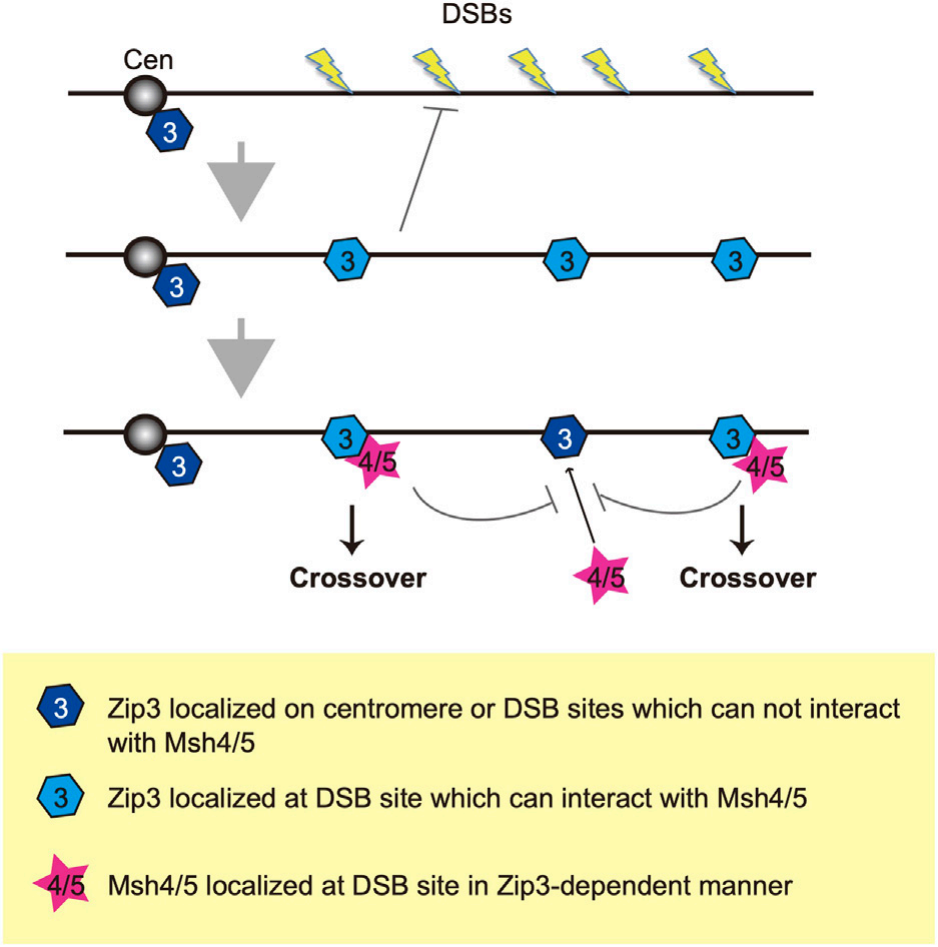
A model of two-step selection of CO formation through Zip3 and Msh4-Msh5. Once DSBs are formed on the chromosome, Zip3 may translocate onto roughly selected DSB sites and suppresses additional DSB formation. There are two kinds of modes of Zip3: One is localized at the DSB site and can recruit Msh4-Msh5 (pale blue) and another cannot recruit Msh4-Msh5 (dark blue). Then, the Msh4-Msh5 complex is recruited to the pro-CO site in a Zip3-dependent manner. Zip3 and Msh4-Msh5 suppress additional complex formation in a coordinated manner. Cen indiate a centromere.

We propose two distinct homeostatic responses to the assembly of ZMM proteins to DSBs (Figure 6). First, DSB formation and/or associated regulatory mechanisms control the number of ensembles containing ZMM core proteins including Zip3. Second, a subset of Zip3 ensembles might be converted into ensembles with Msh4/Msh5. This second step is also under the control of DSB responses. The double-staining analysis of Zip3 with Msh5 (Figure 3B) supports the presence of two populations of Zip3 foci on meiotic chromosomes. Zip3 foci associated with Msh5 show a homeostatic response to DSBs and become brighter relative to early Zip3 foci (Figure 3A; Supplementary Figure S3E). This might be positive feedback of Zip3-focus formation once colocalized with Msh5. The first step seems to be sensitive to the N-terminal region of Xrs2 with the FHA domain. This region is critical for Tel1 (ATM)-mediated phosphorylation of Hop1-pT318 on meiotic chromosomes in the *rad50S* background (Iwasaki et al., 2016). Tel1 is shown to control a feedback mechanism of meiotic DSB formation (Anderson et al., 2015; Garcia et al., 2015). The *xrs2* mutation-specific effect of Zip3 homeostasis might be related to the Tel1 function, which should be studied in the future.

Msh5 foci show homeostatic response to reduced DSB levels, particularly in the *xrs2–314M* (∼80% DSB level) and *xrs2–228M* mutants (∼60% DSB level), which also show robust CO homeostasis. This suggests that Msh5, thus, the Msh4-Msh5 complex (MutSγ) is a critical machinery for CO homeostasis. As the Msh4-Msh5 complex stabilized recombination intermediates (Snowden et al., 2004; Cannavo et al., 2020; Kulkarni et al., 2020), it was previously reported that MutSγ recruitment is a critical step in the CO/NCO decision and for CO interference (Bishop and Zickler, 2004; Snowden et al., 2004; Stahl et al., 2004; Shinohara et al., 2008). The MutSγ complex seems to be a key effector for CO control during meiosis. Alternatively, the complex is a downstream readout for the control.

In mouse spermatocytes, MutSγ foci persist longer in late zygotene/early pachytene stages relative to RAD51/DMC1 foci (Moens et al., 2002). Moreover, the number of MSH4-MSH5 foci is less than RAD51/DMC1 foci but is more than MLH1-MLH3 (MutLγ) foci, suggesting the step-wise implementation of ZMM foci for CO formation/control during mouse meiotic prophase I (Reynolds et al., 2013; Qiao et al., 2014). Similarly in *Sordaria*, MSH4 foci appear in early meiotic prophase than MLH1 foci and the number of MSH4 is higher than that of MLH1 (Storlazzi et al., 2010). Interestingly, a recent study showed a chromosomal localization of a tagged version of Mlh1 in the budding yeast and the number of Mlh1 foci is less than ZMM foci in wild type, supporting a regulatory transition from MutSγ to MutLγ is operating in yeast meiosis (Sanchez et al., 2020).

Prior to Msh4-Msh5 assembly, Zip3 is recruited to chromosomes and promotes the assembly of Msh4-Msh5 (Shinohara et al., 2008). We found that 94% of Msh5 foci contained Zip3 foci (Figure 3). A constant Msh5 foci (42-45) level was maintained in each nucleus when CO homeostasis was functioning (Figures 2, 3). The Msh5-Zip3 colocalization frequency was reduced, however, when CO homeostasis was compromised, i.e., in *xrs2–84M* mutant cells. We hypothesize that Zip3-dependent recruitment of Msh4-Msh5 complexes to DSB sites is critical in CO homeostasis and interference. Again, in both mouse spermatocytes and *Sordaria* meiosis, Zip3 orthologues, Rnf212 and Hei10 (and also Mer3/Hfm1 foci) appear earlier than Msh4 foci (Reynolds et al., 2013; Qiao et al., 2014; De Muyt et al., 2018; Dubois et al., 2019). Thus, it is likely that a Zip3-dependent assembly of the Msh4-Msh5 complex in CO formation/control is evolutionarily conserved.

Previous cytological studies on ZMM foci such as Zip3 revealed that Zip3 foci are evenly spaced along chromosome axes (Fung et al., 2004; Zhang et al., 2020). Based on these, the establishment of the CO designation may occur prior to ZMM assembly. Since the number of Msh5 foci maintain on chromosomes when DSB frequencies are reduced, Msh5-mediated CO homeostasis might operate after the CO designation thus, CO interference and/or CO assurances.

In this study, we observed homeostatic responses to ZMM protein to reduced DSBs in a context-dependent manner, which includes a type of mutant, a tag to the protein, and antibodies or a combination of antibodies used for the immuno-staining. Thus, we need a more careful evaluation of the conclusion obtained by cytological analyses.

### CO homeostasis varies between a short and a long chromosome

A previous study by Martini et al. (2006) analyzed CO homeostasis on chromosome III (3 intervals), VII (3 intervals), and VIII (2 intervals) in *spo11* mutants and focused on the effect of reduced DSB levels on total COs on all three chromosomes but did not study the chromosome-specific variation of CO homeostasis in detail. In this study, by analyzing one more additional interval in both chromosome III and VII, we not only confirmed “global” CO homeostasis but also examined the chromosome-specific effect on CO homeostasis. In CO homeostasis, the relatively short chromosome III was more sensitive to DSB reductions than the longer chromosome VII (Figure 4C).

In the wild type, five of seven intervals on chromosome III (*URA3–LEU2*, *HIS4–MAT*, *HML–LEU2*, *URA3–HIS4*, and *LEU2–MAT*) were genetically longer than the *MET13*–*CYH2* interval (12 cM) on chromosome VII (Figure 2B; Supplementary Tables S2, S3). CO frequency associated with the *MET13*–*CYH2* interval was only mildly affected as DSBs were reduced to ∼20% (in the *xrs2–84M* mutant). In contrast, the five intervals on chromosome III showed significant reductions in CO frequencies (*p* < 0.001) in the *xrs2–84M* mutant. This suggested that chromosome III is more sensitive to DSB reductions than chromosome VII. CO homeostasis likely works in a long chromosome better than a short chromosome. Alternatively, given that, together with the two shortest chromosomes I and VI, chromosome III is unique in the regulation of DSB formation (Murakami et al., 2020), rather than chromosome length by itself, the chromosome-specific property may determine the level of CO homeostasis.

We observed reduced and increased CO frequencies in *MET13–CYH2* and *CYH2–TRP5,* respectively, in the three *xrs2* mutants. These intervals previously analyzed in *spo11* hypomorphic mutants (Martini et al., 2006) exhibited similar tendencies. This suggests that different intervals exhibit different sensitivities or responses to reduced frequencies of DSBs, even for intervals on the same chromosome. In addition, we found that the *xrs2–314M* mutant (∼65% DSBs) showed weakened CO homeostasis in two intervals that spanned a centromere, *HIS4–MAT* and *TRP5–ADE6* compared to other intervals, which is consistent with the suggestion that centromeres may represent a barrier for CO homeostasis, as has been suggested (Martini et al., 2006).

### COs were maintained at the expense of NCO, and reduced level of DSBs weakened CO interference

NCOs tend to be more sensitive to DSB reductions than COs in a manner that is independent of chromosome size. This is particularly seen in the *xrs2–228M* mutant (∼65% DSBs), which showed reduced NCO frequencies at 9 loci while maintaining wild-type levels of COs. COs may be maintained at the expense of NCOs, roughly as proposed (Martini et al., 2006). On the other hand, CO and NCO formation showed similar responses to severe reductions in meiotic DSBs (i.e., they did not simply compensate for one another in the case of *xrs2–84M* mutant), suggesting that CO and NCO were controlled through different mechanisms, consistent with previous reports that NCOs differentiate earlier than CO in DSB processing (Allers and Lichten, 2001; Hunter and Kleckner, 2001). Moreover, these suggested that, in CO homeostasis, certain thresholds of DSBs might upregulate meiotic CO formation within each chromosome or genetic interval.

Although CO interference function even when DSBs were reduced by 80% (*xrs2–84M* mutant), we observed CO interference with reduced its strength. Moreover, for the *URA3–LEU2–HIS4* interval on chromosome III, no CO interference was seen in the *xrs2–84M* mutant (Figure 5B). In addition, the non-Mendelian fraction associated with CO (CRA class) at this locus showed reduced CO homeostasis in the *xrs2–84M* mutant (Figure 4F). This suggested that there might be a coordinating mechanism between CO interference and CO homeostasis, as well as DSB formation.

We note a remarkable difference between reductions in the relative ratio of Msh5 foci and CO frequency, which dropped to 38.6% and 73.6% of wild type, respectively, in the *xrs2–84M* mutant (Figure 2B, 4C). One possibility is that reduced DSB frequencies may stimulate ZMM-independent CO formation pathway(s) that are out of CO interference regulation (Sym et al., 1993; Shinohara et al., 2008). SC elongation is required for the downregulation of meiotic DSB formation (Xu et al., 1995; Tung et al., 2000; Carballo et al., 2013; Gray et al., 2013; Rockmill et al., 2013). The completion of SC elongation may provide a signal that there are sufficient DSBs to generate COs and control CO formation. In contrast, incomplete SC elongation may promote additional meiotic DSB formation which may result in the formation of non-interfering COs (Lee et al., 2021). SC elongation may be involved in CO homeostasis by regulating DSB formation (and non-interfering CO). In the *xrs2–84M* mutant, which had 76% fewer DSBs of the wild type, elongation of Zip1 was severely reduced, whereas CO interference still functions, albeit at reduced effectiveness. This indicated that Zip1 elongation was not critical for CO interference as proposed previously (Zhang et al., 2014).

## Data Availability

The original contributions presented in the study are included in the article/Supplementary Material, further inquiries can be directed to the corresponding author.

## Supplementary material

SUPPLEMENTARY FIGURE S1 Rad51 focus formation in the *spo11* hypomorph mutants. **(A)** Y-axis showed an average number of Rad51 foci in nuclei in wild type (100%), *spo11-HA*/*spo11-HA* (80%), *spo11-HA*/*spo11-YF* (30%), and *spo11-YF*/*spo11-YF* (0%). Values of the X-axis were referred from Martini *et al.* (Martini et al., 2006). Error bars indicate standard deviations from independent trials (left). The distribution of the number of Rad51 foci in wild-type and *spo11* hypomorph mutants (raw values) is shown. Error bars show medians and interquartile ranges. **(B)** The distributions of the number of Zip3 and Msh5 foci in wild-type and *spo11* hypomorph mutants are shown. Error bars show medians and interquartile ranges. The numbers at the top of the graph indicate the median value. The statistical significance of differences was determined using the Mann-Whitney *U*-test (*****p* < 0.0001). **(C)** The average number of Zip3 and Msh5 foci plotted in each *spo11* mutant strain (left). Values are presented as a ratio relative to the wild type against each relative number of Rad51 foci [i.e., DSBs, **(A)**] in each strain (right).

SUPPLEMENTARY FIGURE S2 Hop1 localization in the BRCT-like domain-deleted *xrs2* mutant Localization of Zip1 (red; rabbit) and Hop1 (green; guinea pig) in wild type, *xrs2-314M*, and *pch2* mutant cells are shown. Scale bar = 2 µm.

SUPPLEMENTARY FIGURE S3 Comparison of focus number of Zip3 and Msh5 in various *xrs2* mutants. **(A)** The number of foci of Zip3 and Msh5 per nucleus in different time points after meiosis entry in wild type, *xrs2-84M*, *xrs2-228M*, and *xrs2-314M.* Error bars show medians and interquartile ranges. **(B)** The number of foci of Zip3 and Msh5 per nucleus from Figure 2B was shown in a scatter dot plot. The number of nuclei analyzed for Figure 2; wild type, *xrs2-84M*, *xrs2-228M*, and *xrs2-314M* are 459, 416, 542, and 339, respectively (Zip3 foci), and 454, 338, 345, and 219, respectively (Msh5 foci). Error bars show medians and interquartile ranges. **(C)** The number of Zip3- and Msh5-foci from co-localization analysis shown in Figure 3 is shown in a scatter dot plot. Error bars show medians and interquartile ranges. **(D)** Kinetics of frequency of Zip3- and Msh5-focus positive nuclei in wild type, *xrs2–228M*, and *xrs2–84M* during meiosis. Error bars show the average and SD. **(E)** The fluorescent signal intensity of Zip3 foci colocalized with or without Msh5 foci was measured and plotted. The *p*-value was calculated with Mann-Whitney *U*-test. Note the number of Zip3 alone foci is smaller than that of Zip3 foci with Msh5 (Figure 3B).
