# Supplementary figures and images for: The Msh5 complex shows homeostatic localization in response to DNA double-strand breaks in yeast meiosis
Source: Front Cell Dev Biol. 2023 May 18;11:1170689. doi: 10.3389/fcell.2023.1170689 (PMC10232913; doi:10.3389/fcell.2023.1170689)

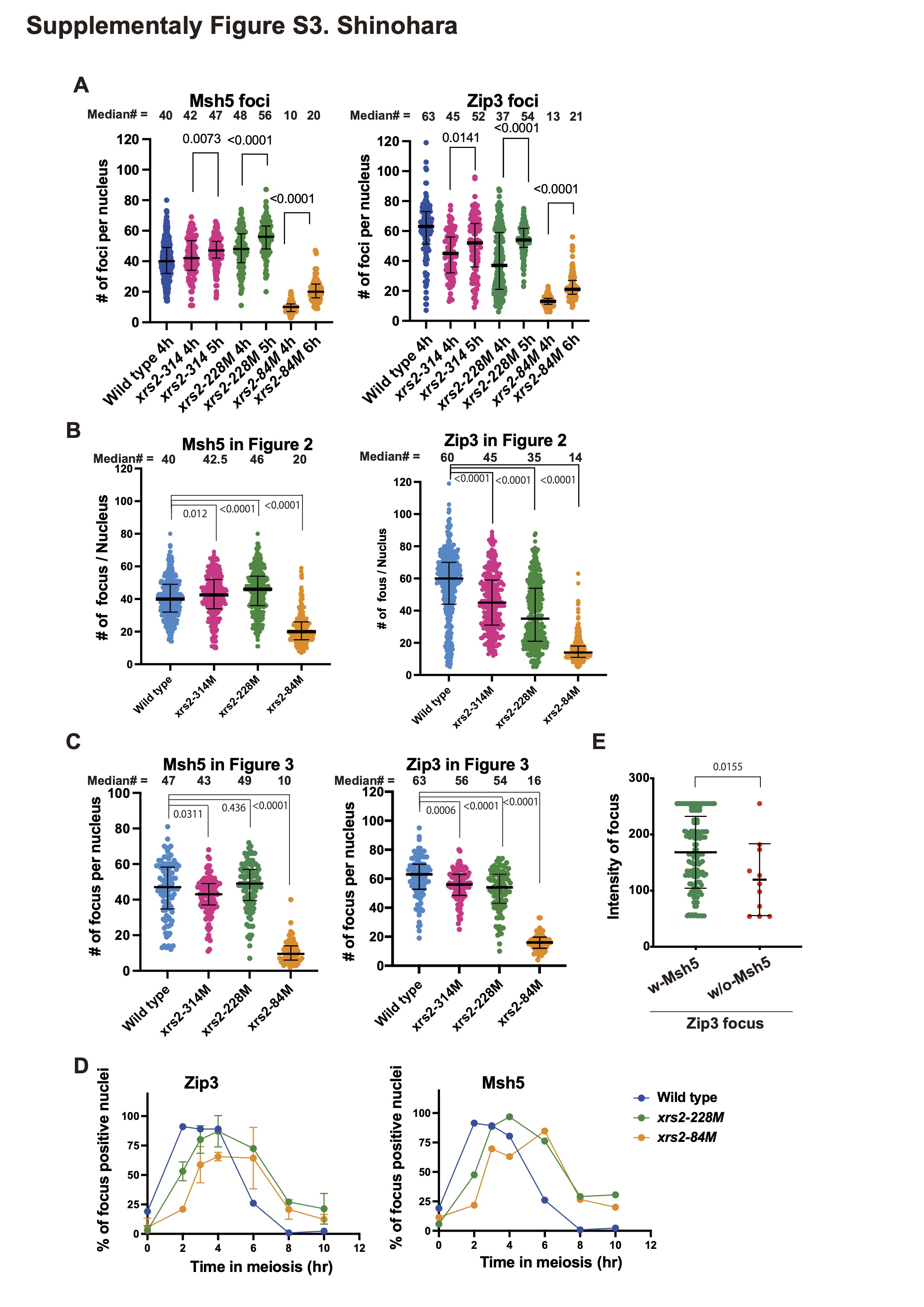

Supplement: Supplementary file 1 [file Image3.JPEG]

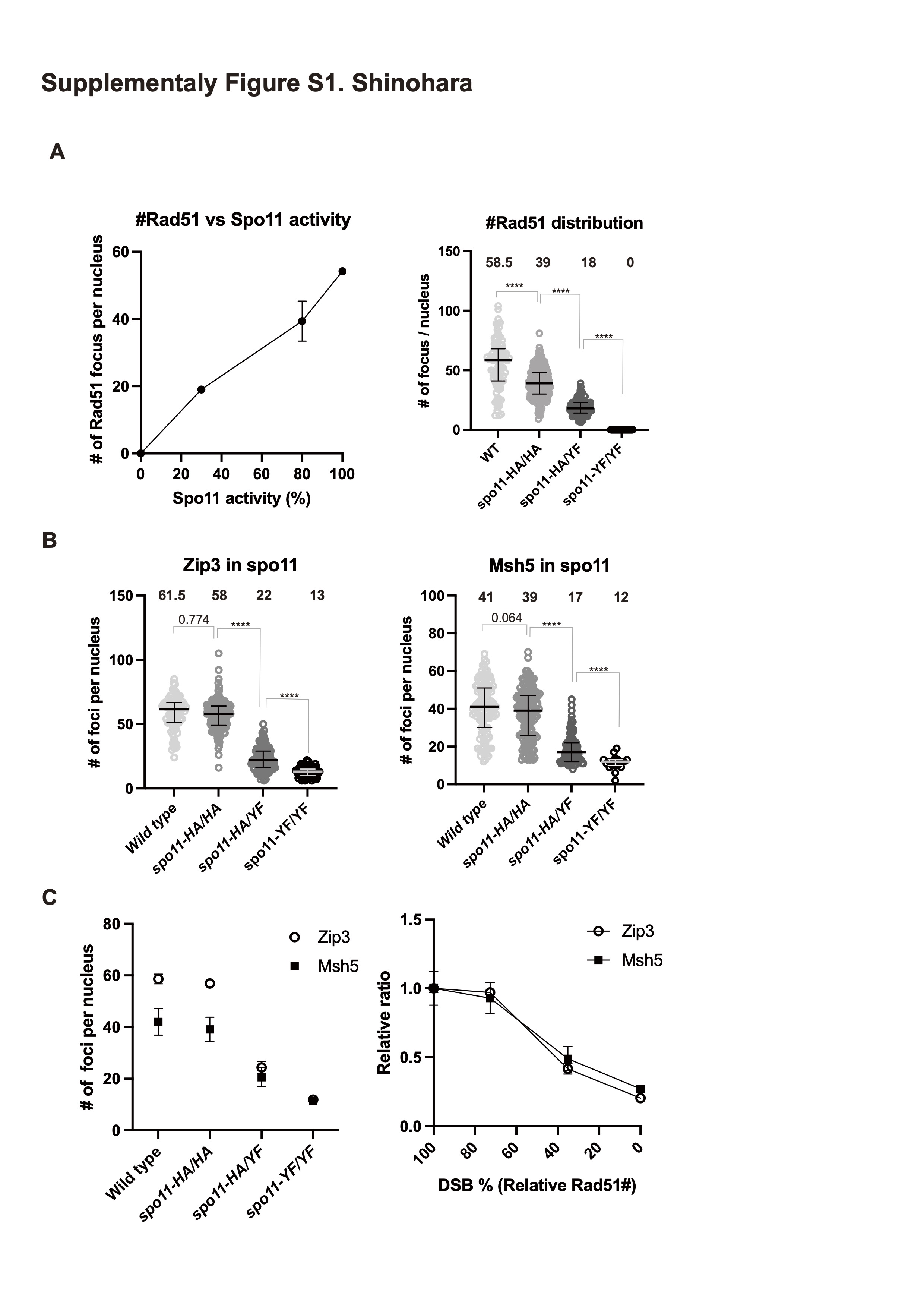

Supplement: Supplementary file 2 [file Image1.JPEG]

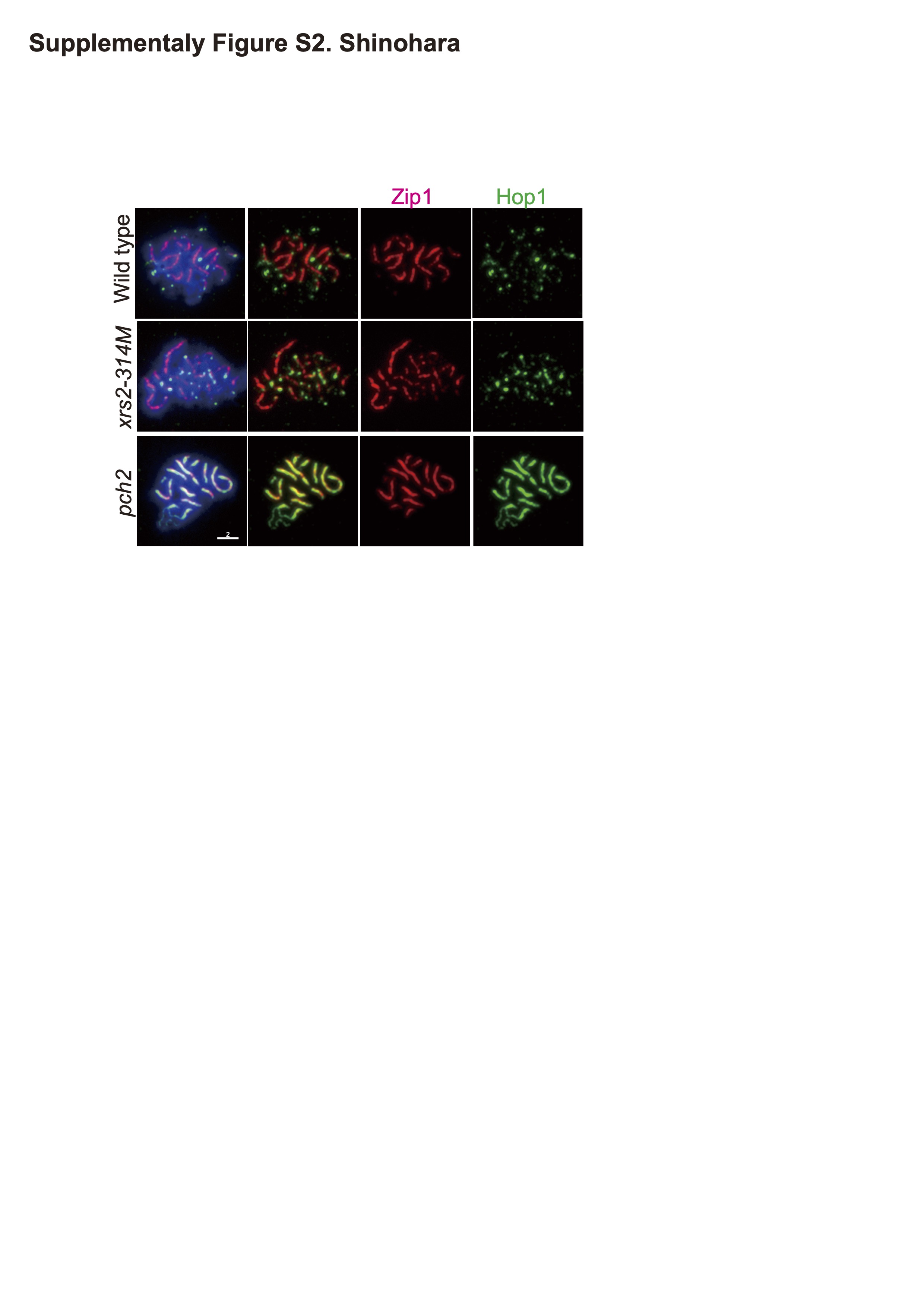

Supplement: Supplementary file 3 [file Image2.JPEG]
