# Supplementary material for: The Msh5 complex shows homeostatic localization in response to DNA double-strand breaks in yeast meiosis
Source: Front Cell Dev Biol. 2023 May 18;11:1170689. doi: 10.3389/fcell.2023.1170689 (PMC10232913; doi:10.3389/fcell.2023.1170689)
Supplement: Supplementary file 4 [file DataSheet1.PDF]

Supplemental Table-1 Strain list

Strain	Genotype	Reference
NKY1551	*MATa*/a, *ho::LYS2/", lys2/", ura3/", leu2::hisG/", his4X-LEU2(Bam*HI*)-URA3/his4B-LEU2(Mlu*I*), arg4-nsp/arg4-bgl*	(Storlazzi et al., 1996)
MSY1992	NKY1551 with *xrs2-314M*	(Shima et al., 2005)
MSY1524	NKY1551 with *xrs2-228M*	(Shima et al., 2005)
MSY1494	NKY1551 with *xrs2-84M*	(Shima et al., 2005)
MSY6446/6448	NKY1551 with *pch2::TRP1*	This study
MSY833/831	*MATa*/a, *ho::LYS2/", lys2/", ura3/", leu2::hisG/", trp1::hisG/"*	(Shinohara et al., 2015)
MSY4177	MSY833/831 with *spo11-3xHA::KANMX*/"	This study
MSY4185	MSY833/831 with *spo11-3xHA::KANMX/spo11-Y135F-3xHA::KANMX*	This study
MSY4189	MSY833/831 with *spo11-Y135F-3xHA::KANMX*/"	This study
MSY4304	a, *ho::LYS2, lys2, his4B-leu2-E*, *cup2-B*, *met13-B*, *trp5-S*, *ade6-B*, *arg4-bgl*	(Lee et al., 2021)
MSY4245	*a*, *ho::LYS2, lys2, hml::KanMX6*, *HIS4-LEU2-URA3*, *cyh2-R*, *arg4-bgl*	(Lee et al., 2021)
MSY4314	MSY4304 with *xrs2-314M*	This study
MSY4316	MSY4245 with *xrs2-314M*	This study
MSY4310	MSY4304 with *xrs2-228M*	This study
MSY4312	MSY4245 with *xrs2-228M*	This study
MSY4306	MSY4304 with *xrs2-84M*	This study
MSY4308	MSY4245 with *xrs2-84M*	This study

Lee, M.S., Higashide, M.T., Choi, H., Li, K., Hong, S., Lee, K., et al. (2021). The

synaptonemal complex central region modulates crossover pathways and feedback control of meiotic double-strand break formation. *Nucleic Acids Res* 49(13), 7537-7553. doi: 10.1093/nar/gkab566.

Shima, H., Suzuki, M., and Shinohara, M. (2005). Isolation and characterization of novel xrs2 mutations in Saccharomyces cerevisiae. *Genetics* 170(1), 71-85. doi: 10.1534/genetics.104.037580.

Shinohara, M., Hayashihara, K., Grubb, J.T., Bishop, D.K., and Shinohara, A. (2015). DNA damage response clamp 9-1-1 promotes assembly of ZMM proteins for formation of crossovers and synaptonemal complex. *J Cell Sci* 128(8), 1494-1506. doi: 10.1242/jcs.161554.

Storlazzi, A., Xu, L., Schwacha, A., and Kleckner, N. (1996). Synaptonemal complex (SC) component Zip1 plays a role in meiotic recombination independent of SC polymerization along the chromosomes. *Proc Natl Acad Sci U S A* 93(17), 9043-9048.

Supplemental Table 2. Genetical analysis of *xrs2* mutant cells by Papazian's NPD analysis on chromosome VII

Strain (Total # of tetrad)	Intervals	PD	TT	NPD_{obs}	Total	NPD_{exp}	cM (S.E.) [a]	NPD ratio (S.D.) [b]	*P*-value [c]	*P*-value [d]
Wild type (N=1200)	*CUP2-MET13*	495	551	18	1064	61	31.0+/-1.45	0.30+/-0.07	<0.0001	
	MET13-CYH2	854	265	0	1119	9	11.8+/-1.82	<0.11	0.386	
	CYH2-TRP5	387	723	40	1150	128	41.9+/-382	0.31+/-0.06	<0.0001	
	TRP5-ADE6	298	756	92	1146	168	57.1+/-2.32	0.55+/-0.09	<0.0001	
	CUP12-CYH2	364	694	32	1090	129	40.6+/-5.08	0.25+/-0.05	<0.0001	
	MET13-TRP5	292	754	71	1117	151	52.8+/-5.05	0.47+/-0.05	<0.0001	
	CYH2-ADE6	210	774	158	1142	147	75.4+/-563	1.08+/-0.14	0.415	
xrs2-314M (N=1200)	*CUP2-MET13*	456	583	25	1064	72	34.4+/-3.07	0.35+/-0.07	<0.0001	0.062
	MET13-CYH2	884	250	1	1135	8	11.3+/-1.69	0.12+/-0.12	<0.0001	0.405
	CYH2-TRP5	361	743	65	1169	137	48.5+/-4.20	0.47+/-0.07	<0.0001	0.030
	TRP5-ADE6	316	754	80	1150	161	53.7+/-5.68	0.50+/-0.08	<0.0001	0.270
	CUP12-CYH2	342	702	57	1101	131	47.4+/-1.65	0.43+/-0.07	<0.0001	0.044
	MET13-TRP5	263	782	82	1127	106	56.5+/-0.70	0.78+/-0.09	0.059	0.098
	CYH2-ADE6	206	785	165	1156	145	76.8+/-8.56	1.14+/-0.15	0.140	0.670
xrs2-228M (N=1367)	*CUP2-MET13*	423	750	38	1211	127	40.4+/-2.31	0.30+/-0.05	<0.0001	<0.0001
	MET13-CYH2	1010	244	1	1255	7	10+/-1.90	0.15+/-0.15	0.545	0.016

	CYH2-TRP5	349	919	75	1343	152	51+/-2.49	0.50+/-0.07	<0.0001	<0.0001
	TRP5-ADE6	449	815	67	1331	133	45.7+/-1.22	0.50+/-0.04	<0.0001	<0.0001
	CUP12-CYH2	349	884	68	1301	162	49.7+/-1.80	0.42+/-0.03	<0.0001	<0.0001
	MET13-TRP5	270	878	103	1251	99	59.8+/-3.68	1.05+/-0.13	0.762	0.0033
	CYH2-ADE6	255	900	176	1331	176	73.5+/-4.09	1.00+/-0.12	>0.99	0.545
xrs2-84M	*CUP2-MET13*	498	654	38	1190	81	37.1+/-2.32	0.47+/-0.08	0.002	0.007
(N=1275)	*MET13-CYH2*	1031	182	1	1214	4	7.7+/-0.87	0.26+/-0.26	0.740	<0.0001
	CYH2-TRP5	431	751	74	1256	114	47.6+/-3.33	0.65+/-0.09	0.006	0.435
	TRP5-ADE6	583	606	50	1239	60	36.6+/-3.03	0.83+/-0.13	0.486	<0.0001
	CUP12-CYH2	444	729	63	1236	107	44.8+/-1.13	0.59+/-0.09	0.002	0.87
	MET13-TRP5	368	747	95	1210	125	54.4+/-4.87	0.76+/-0.11	0.037	0.0229
	CYH2-ADE6	297	798	144	1239	156	67.1+/-4.13	0.93+/-0.14	0.410	0.001

Map distances and NPD ratio were calculated as describes in Experimental procedures. NPDobs is a number of NPD observed in crosses. NPDexp is a number of NPD expected from Papazian equation.

a Standard error of map distances was calculated from four independent crosses.

b Standard error of NPD ratio was calculated using the Stahl Lab online tool (https://elizabethhousworth.com/StahlLabOnlineTools/).

c Statistically significant differences between the expected and observed tetrad type in each strain were assessed using Chi-square test using GraphPad Prism9.

c Statistically significant differences between the wild type and each strain were assessed using Chi-square test using GraphPad Prism9.

Supplemental Table 3. Genetical analysis of *xrs2* mutant cells by Papazian's NPD analysis on chromosome III

Strain	Intervals	PD	TT	NPD_{obs}	Total	NPD_{exp}	cM (S.E.)[a]	NPD ratio (S.D.)[b]	*P*-value[c]
Wild type	*HML-URA3*	960	208	1	1169	5	9.2+/-1.04	0.19+/-0.19	0.674
	URA3-LEU2	812	296	4	1112	12	14.4+/-1.00	0.33+/-0.17	0.466
	LEU2-HIS4	1079	41	0	1120	0	1.8+/-0.33	N.A.	N.A.
	HIS4-MAT	412	709	45	1166	113	42+/-0.73	0.40+/-0.07	<0.0001
	HML-LEU2	651	459	8	1118	34	22.7+/2.28	0.23+/-0.08	0.039
	URA3-HIS4	800	360	5	1165	18	16.7+/-0.94	0.28+/-0.13	0.272
	LEU2-MAT	375	689	47	1111	117	43.7+/-0.41	0.40+/-0.07	<0.0001
	HML-HIS4	641	519	12	1172	43	25.2+/-2.03	0.28+/-0.08	0.019
xrs2-314M	*HML-URA3*	1039	131	2	1172	2	6.1+/-0.79	1.0+/-0.71	>0.99
	URA3-LEU2	722	382	7	1111	22	19.1+/-1.93	0.32+/-0.12	0.002
	LEU2-HIS4	1064	51	0	1115	0	2.3+/-1.43	N.A.	N.A.
	HIS4-MAT	441	682	44	1167	98	40.5+/-1.33	0.45+/-0.08	0.211
	HML-LEU2	618	487	13	1118	40	25.3+/-2.59	0.33+/-0.09	0.036
	URA3-HIS4	696	457	10	1163	32	22.2+/-4.01	0.31+/-0.10	0.085
	LEU2-MAT	389	678	45	1112	109	42.6+/-1.89	0.41+/-0.07	<0.0001
	HML-HIS4	592	562	16	1170	54	28.1+/-4.65	0.30+/-0.08	<0.0001
xrs2-228M	*HML-URA3*	1216	133	0	1349	2	4.9+/-0.71	<0.57	0.800

	URA3-LEU2	849	431	13	1293	24	19.6+/-1.61	0.55+/-0.15	0.400
	LEU2-HIS4	1276	26	0	1302	0	1.0+/-0.27	N.A.	N.A.
	HIS4-MAT	602	697	48	1347	77	36.5+/-1.4	0.63+/-0.10	0.052
	HML-LEU2	749	541	15	1305	41	24.1+/-1.85	0.37+/-0.10	0.059
	URA3-HIS4	847	481	14	1342	29	21.02.50	0.48+/-0.10	0.265
	LEU2-MAT	565	680	53	1298	77	38.4+/-2.05	0.69+/-0.10	0.104
	HML-HIS	746	592	16	1339	186	25.4+/-2.53	0.46+/-0.04	<0.0001
xrs2-84M	*LEU2-MAT*	1184	76	0	1260	1	3+/-0.32	<1.68	0.869
	URA3-LEU2	1072	158	0	1230	3	6.4+/-1.09	<0.35	0.722
	LEU2-HIS4	1188	47	0	1235	0	1.9+/-0.76	N.A.	N.A.
	HIS4-MAT	702	525	28	1237	40	27.6+/-2.24	0.70+/-0.14	0.887
	HML-LEU2	1003	233	1	1237	6	9.7+/-1.29	0.16+/-0.16	0.618
	URA3-HIS4	1044	209	0	1253	10	11.5+/-0.43	<0.20	0.306
	LEU2-MAT	646	557	28	1231	48	29.4+/-2.37	0.58+/-0.11	0.149
	HML-HIS4	976	284	1	1261	10	11.5+/-0.62	0.11+/-0.11	0.408

Map distances and NPD ratio were calculated as describes in Experimental procedures. NPDobs is a number of NPD observed in crosses. NPDexp is a number of NPD expected from Papazian equation.

a Standard error of map distances was calculated from four independent crosses.

b Standard error of NPD ratio was calculated using the Stahl Lab online tool (https://elizabethhousworth.com/StahlLabOnlineTools/).

c Differences between the expected and observed tetrad type in each strain were calculated using the Stahl Lab online tool.

Supplemental table 4; Non-Mendel segregation frequencies on chromosome III and chromosome VII

Strain	Chrosome III					**Chromosome VII**				
	HML	*URA3*	*LEU2*	*HIS4*	*MAT*	*CUP2*	*MET13*	*CYH2*	*TRP5*	*ADE6*
Wild type	1.4 (1.0)	2.3 (1.0)	6.3 (1.0)	1.7 (1.0)	1.8 (1.0)	7.8 (1.0)	5.8 (1.0)	2.8 (1.0)	2.0 (1.0)	3.3 (1.0)
xrs2-314M	1.4 (1.0)	2.1 (0.9)	6.3 (1.0)	1.7 (1.0)	1.3 (0.9)	7.3 (0.9)	4.7 (0.8)	1.3 (0.4)	1.8 (0.9)	2.8 (0.8)
xrs2-228M	0.4 (0.3)	1.3 (0.6)	4.3 (0.7)	0.7 (0.4)	0.7 (0.4)	4.2 (0.5)	7.8 (1.3)	1.0 (0.5)	1.0 (0.5)	1.8 (0.5)
xrs2-84M	0.3 (0.2)	1.1 (0.5)	2.7 (0.4)	0.9 (0.5)	0.7 (0.4)	2.6 (0.3)	4.3 (0.8)	0.7 (0.4)	0.8 (0.4)	2.2 (0.7)

Frequencies of tetrad with 3+:1-, 1+:3-, 4+:0-, 0+:4- segregation for each marker were calculated. Relative ratios of the frequencies in the mutant to wild type are shown in parenthesis.

Supplemental Table. 4 Genetical analysis of *xrs2* mutant cells by three factor cross

Chromosome III	Frequency of consecutive crossovers observed			Frequency of consecutive crossovers expected			Ratio (observed/expected)		
Intervals	*HML-URA3-LEU2*	*URA3-LEU2-HIS4*	*LEU2-HIS4-MAT*	*HML-URA3-LEU2*	*URA3-LEU2-HIS4*	*LEU2-HIS4-MAT*	*HML-URA3-LEU2*	*URA3-LEU2-HIS4*	*LEU2-HIS4-MAT*
Wild type	0.047	0.010	0.023	0.016	0.006	0.014	0.348	0.667	0.596
xrs2-314M	0.040	0.015	0.028	0.016	0.007	0.014	0.417	0.484	0.496
xrs2-228M	0.033	0.009	0.015	0.013	0.004	0.011	0.377	0.430	0.752
xrs2-84M	0.008	0.005	0.017	0.005	0.005	0.009	0.637	1.011	0.535

Chromosome VII	Frequency of consecutive crossovers observed			Frequency of consecutive crossovers expected			Ratio (observed/expected)		
Intervals	*CUP2-MET13-CYH2*	*MET13-CYH2-TRP5*	*CYH2-TRP5-ADE6*	*CUP2-MET13-CYH2*	*MET13-CYH2-TRP5*	*CYH2-TRP5-ADE6*	*CUP2-MET13-CYH2*	*MET13-CYH2-TRP5*	*CYH2-TRP5-ADE6*
Wild type	0.124	0.155	0.606	0.084	0.131	0.606	0.673	0.848	1.000
xrs2-314M	0.135	0.166	0.498	0.089	0.122	0.485	0.656	0.734	0.974
xrs2-228M	0.121	0.141	0.480	0.094	0.124	0.464	0.779	0.877	0.965
xrs2-84M	0.088	0.098	0.343	0.075	0.090	0.347	0.862	0.922	1.012
